# Mutation of *PTPN11* (Encoding SHP-2) Promotes MEK Activation and Malignant Progression in Neurofibromin-Deficient Cells in a Manner Sensitive to *BRAP* Mutation

**DOI:** 10.3390/cancers14102377

**Published:** 2022-05-12

**Authors:** Ritsuko Harigai, Ryo Sato, Chikako Hirose, Toshiki Takenouchi, Kenjiro Kosaki, Takanori Hirose, Hideyuki Saya, Yoshimi Arima

**Affiliations:** 1 Division of Gene Regulation, Institute for Advanced Medical Research, Keio University School of Medicine, 35 Shinano-machi, Shinjuku-ku, Tokyo 160-8582, Japan; harigai106@yahoo.co.jp (R.H.); sator3@nih.gov (R.S.); miyazaki.chikako@gmail.com (C.H.); hsaya@a5.keio.jp (H.S.); 2 Department of Pediatrics, Keio University School of Medicine, 35 Shinano-machi, Shinjuku-ku, Tokyo 160-8582, Japan; toshiki.take@keio.jp; 3 Center for Medical Genetics, Keio University School of Medicine, 35 Shinano-machi, Shinjuku-ku, Tokyo 160-8582, Japan; kkosaki@keio.jp; 4 Department of Pathology for Regional Communication, Kobe University Graduate School of Medicine, 7-5-1 Kusunoki-cho, Chuo-ku, Kobe 650-0017, Japan; thirose@hp.pref.hyogo.jp

**Keywords:** neurofibromatosis type 1 (*NF1*), *PTPN11*, SHP-2, *BRAP*, MEK

## Abstract

**Simple Summary:**

Germline mutations of *NF1* cause neurofibromatosis type 1 (*NF1*), which is characterized by multiple benign peripheral nerve sheath tumors known as neurofibromas. In some individuals with *NF1*, plexiform neurofibromas can give rise to malignant peripheral nerve sheath tumors. Here, we applied genomic DNA sequencing to *NF1*-derived tumors and identified additional genetic alterations in *PTPN11* (encoding Src homology region 2 domain-containing phosphatase-2 (SHP)-2) and *BRAP* associated with *NF1* tumor malignancy. We found that the forced expression of the mutant form of SHP-2 activated the protein kinase MEK and increased tumorigenic activity in *NF1* cells, and that these effects were attenuated by the forced expression of the mutant form of BRCA1-associated protein (*BRAP*). This suppressive action of mutant *BRAP* was not apparent in *NF1*-intact cells. Our data indicate that the combination of *NF1* mutation and *PTPN11* mutation drives the malignancy of *NF1* cells and that SHP-2 inhibition by *BRAP* is a potential therapeutic strategy for *NF1*-associated malignant tumors.

**Abstract:**

Germline mutations of *NF1* cause neurofibromatosis type 1 (*NF1*) through the activation of the *RAS* signaling pathway, and some *NF1* patients develop malignant peripheral nerve sheath tumors (MPNSTs). Here, we established subclones of the human *NF1*-MPNST cell line sNF96.2 that manifest increased tumorigenic activity and increased phosphorylation of the protein kinases MEK and Akt relative to the parental cells. Genomic DNA sequencing identified 14 additional heterozygous mutations within the coding regions of 13 cancer- and other disease-related genes in these subclones. One of these genes, *PTPN11*, encodes SHP-2, and the forced expression of the identified G503V mutant of SHP-2 increased both tumorigenic activity and MEK phosphorylation in parental sNF96.2 cells, suggesting that the combination of *PTPN11* and *NF1* mutations induces the pathological activation of the *RAS* pathway. These effects of SHP-2 (G503V) were inhibited by the coexpression of the G370A mutant of *BRAP*, which was also detected in the highly malignant subclones, and this inhibition was accompanied by the calpain-dependent cleavage of SHP-2 (G503V). The cleavage of SHP-2 (G503V) and suppression of MEK phosphorylation mediated by *BRAP* (G370A) were not detected in *NF1*-intact (HeLa) cells. Tumor promotion by SHP-2 (G503V) and its suppression by *BRAP* (G370A) may serve as a basis for the development of new treatment strategies for *NF1*.

## 1. Introduction

Malignant peripheral nerve sheath tumors (MPNSTs) are soft-tissue sarcomas that surround peripheral nerves and occur either sporadically or more often in association with neurofibromatosis type 1 (*NF1*, von Recklinghausen disease), an inherited disorder caused by germline mutations of the *NF1* gene [1,2]. *NF1* is inherited in an autosomal-dominant manner and affects 1 in ~3000 live births, with 30% to 50% of *NF1* mutations arising de novo [3,4,5]. Individuals with *NF1* manifest a variety of clinical symptoms, including peripheral and central nervous system tumors, pigmentary changes such as multiple café au lait spots, bone defects, cardiovascular abnormalities, and learning disabilities [6]. The development of single or multiple benign peripheral nerve sheath tumors known as neurofibromas is a common symptom of *NF1*. These lesions can occur at virtually any site in the body and are generally divided into nodular plexiform, diffuse plexiform, subcutaneous, and cutaneous subtypes [7,8]. Whereas the life expectancy of individuals with *NF1* is almost normal, the malignant transformation of benign tumors is associated with a reduction in overall survival. Most MPNSTs arise from plexiform neurofibromas, and the overall prognosis of patients with such tumors is poor, with a 5-year survival rate of ~34% to 52% having been reported [9]. Another recent cohort study showed that, among 1607 *NF1* patients, 243 individuals (15.1%) developed MPNSTs, and that the 5-year disease-specific survival rate of these individuals was lower than that of patients with MPNSTs not related to *NF1* (31.6% versus 43.4–71.9%) [10]. A better understanding of the mechanisms underlying the malignant transformation of neurofibromas would be expected to contribute to the development of therapeutic strategies that improve the prognosis of *NF1* patients.

*NF1* mutations have been identified both in *NF1* patients and in individuals with sporadic brain, breast, or lung cancer [11]. *NF1* is a tumor suppressor gene and encodes a 2818-amino acid protein termed neurofibromin that functions as a GTPase-activating protein for the small GTPase *RAS* (*RAS*-GAP) and therefore negatively regulates *RAS* signaling by promoting the conversion of the GTP-bound (active) form of *RAS* to the GDP-bound (inactive) form. The loss or mutation of neurofibromin thus results in the activation of *RAS* and the engagement of its downstream effectors [12,13].

The *RAS*-RAF-MEK-ERK (*RAS*-ERK) signaling pathway is affected in many human cancers as a result of the abnormal activation of upstream receptor tyrosine kinases or gain-of-function mutations predominantly of the *RAS* or *RAF* genes [14,15]. This pathway is thus considered a potential therapeutic target for such cancers [16]. We hypothesize that *NF1* mutation alone is not sufficient to induce the continuous activation of the *RAS*-ERK pathway and that the pathological activation of this pathway promotes the development of *NF1*-associated malignant tumors.

To identify additional genetic alterations that promote the malignancy of *NF1*-associated tumors, we established highly malignant *NF1*-mutated cells and analyzed their genomic DNA using next-generation sequencing. We detected 14 heterozygous variants within the coding regions of 13 cancer- and other disease-related genes, including single-base substitutions in the *PTPN11* (c.1508G > T, p.Gly503Val) and *BRAP* (c.1109G > C, p.Gly370Ala) genes. We examined the effects of these additional mutations on malignant progression in *NF1*-mutated cells.

## 2. Materials and Methods

### 2.1. Cell Lines and Cell Culture

sNF96.2 and HeLa cells were obtained from American Type Culture Collection (ATCC, Manassas, VA, USA) and were maintained in high-glucose DMEM (Nacalai Tesque, Kyoto, Japan) supplemented with 10% fetal bovine serum (FBS). We established sNF96.2 cells that stably expressed green fluorescent protein (GFP), and these sNF96.2-GFP cells were maintained in DMEM supplemented with 10% FBS. sNF96.2 cells stably expressing *BRAP* (G370A), SHP-2 (WT), SHP-2 (G503V), or both SHP-2 (G503V) and either *BRAP* (WT) or *BRAP* (G370A), as well as HeLa cells stably expressing SHP-2 (G503V) with or without *BRAP* (G370A), were cultured in DMEM supplemented with 10% FBS and puromycin (Sigma) at 1 µg/mL or blasticidin (Sigma) at 15 µg/mL.

### 2.2. Cell Transplantation in Mice

Balb/c nu/nu and NOD/SCID immunodeficient mice were obtained from Charles River. All mouse experiments were approved by the ethics committee of Keio University School of Medicine (approval number 13037), and the animals were treated in compliance with the regulations for animal experiments of Keio University School of Medicine. Mice were anesthetized via the intraperitoneal injection of a combination of medetomidine (0.3 mg/kg), midazolam (4.0 mg/kg), and butorphanol (5.0 mg/kg). For subcutaneous transplantation, cells (5 × 10^6^ in 100 µL of phosphate-buffered saline) were injected into the lower flank of 4-week-old female Balb/c nu/nu mice. For kidney subcapsular transplantation, cells (1 × 10^6^ in 10 µL of Matrigel (Corning)) were injected below the kidney capsule of 4-week-old female Balb/c nu/nu mice.

### 2.3. Establishment of sNF96.2-GFP Tumor–Derived Cells

sNF96.2-GFP cells (3 × 10^6^) were mixed with Matrigel and injected below the kidney capsule of immunodeficient (Balb/c nu/nu or NOD/SCID) mice. After 5 to 6 months, the tumor cells were isolated from the developed tumors and cell subclones were established. A-1 and C-1 cells were obtained from Balb/c nu/nu mice at 154 and 176 days, respectively, after cell injection. B-1 and D-1 cells were obtained from NOD/SCID mice at 163 and 191 days, respectively. These cell subclones were maintained in DMEM supplemented with 10% FBS.

### 2.4. STR Profile Analysis

The short tandem repeat (STR) profiles of sNF96.2-GFP and A-1 cells were determined using polymerase chain reaction (PCR) analysis as performed by the Human Cell Line Authentication Service (Promega, Madison, WI, USA).

### 2.5. Genomic DNA Analysis

Genomic DNA of sNF96.2-GFP, A-1, B-1, C-1, and D-1 cells was analyzed using whole-exome sequencing. The sequence reads were aligned to the reference genome assemblies (hg19) with the use of BWA [17]. MuTect version 1.14 [18] was applied for comparison of the exome data derived from the parental sNF96.2-GFP cells with those derived from A-1, B-1, C-1, and D-1 cells. The default parameters were adopted with the exceptions that max_alt_alleles_in_normal_count and minimum_mutation_cell_fraction were set to 0 and 0.1, respectively.

### 2.6. Plasmids and Gene Transfer

For the establishment of cells stably expressing *BRAP* (G370A), SHP-2 (WT), SHP-2 (G503V), or both SHP-2 (G503V) and either *BRAP* (WT) or *BRAP* (G370A), we inserted human *BRAP*, *BRAP* (c.1109G > C, p.Gly370Ala), *PTPN11*, or *PTPN11* (c.1508G > T, p.Gly503Val) cDNAs into the pMXs-IRES-Puro (pMXs-IP), pMXs-IRES-Blasticidin (pMXs-IB), or pMXs-IRES-GFP (pMXs-IG) retroviral expression vectors (Addgene, Watertown, MA, USA). Plat-A retroviral packaging cells were transfected with the retroviral vectors with the use of the FuGENE HD reagent (Roche, Basel, Switzerland) in order to generate recombinant retroviruses. The culture medium was changed after 24 h, and the virus-containing culture supernatant was collected after culture for an additional 48 h and was passed through a 0.45 µm filter. Each supernatant was added to a RetroNectin dish (TaKaRa Bio, Shiga, Japan) to allow the attachment of the virus particles, and after 6 h, the supernatant was removed and sNF96.2 or HeLa cells were transferred to the dish. The sNF96.2 cells stably expressing *BRAP* (G370A), SHP-2 (WT), SHP-2 (G503V), or both SHP-2 (G503V) and either *BRAP* (WT) or *BRAP* (G370A) and the HeLa cells stably expressing SHP-2 (G503V) with or without *BRAP* (G370A) were selected in high-glucose DMEM supplemented with 10% FBS and either puromycin at 1 µg/mL or blasticidin at 15 µg/mL. The cells stably expressing GFP were sorted via fluorescence-activated cell sorting with a MoFlo XDP Cell Sorter (Beckman Coulter, Brea, CA, USA) on the basis of GFP fluorescence. The transient transfection of HeLa cells with expression vectors (pMXs-IP or pMXs-IG) for SHP-2 (G503V), *BRAP* (G370A), or both SHP-2 (G503V) and *BRAP* (G370A) was performed with the use of the Lipofectamine 2000 reagent (Invitrogen, Waltham, MA, USA) according to the modified protocol proposed by the manufacturer.

### 2.7. RNA Interference

*NF1* and negative control (*GAPD*) small interfering RNAs (siRNAs) were obtained from Dharmacon and were introduced into cells via transfection with the use of the Lipofectamine RNAiMAX reagent (Invitrogen).

### 2.8. Trypan Blue Staining and Cell Viability Assay

For the evaluation of live cell number, cells (2 × 10^4^ per well) were seeded in six-well plates, cultured for the indicated times, and then stained with trypan blue. For the evaluation of cell viability, cells (1 × 10^3^ per well) were seeded in 96-well plates, incubated for 24 h before exposure to trametinib or SHP099 for 72 h, and then assayed for viability with the use of a Cell TiterGlo assay (Promega).

### 2.9. Inhibitors

Trametinib (MEK inhibitor) was obtained from ChemScene, SHP099 (SHP-2 inhibitor) from MedChem Express, and calpeptin (cell-penetrating calpain inhibitor) from Calbiochem.

### 2.10. Immunoblot Analysis

Immunoblot analysis was performed as previously described [19] with primary antibodies to phosphorylated Akt (#4060), to Akt (#9272), to phosphorylated ERK1/2 (#4370), to ERK1/2 (#9102), to phosphorylated MEK (#9121), to MEK (#9122), to phosphorylated S6 (#4858), to S6 (#2217), to phosphorylated STAT3 (#9131), to STAT3 (#4904), and to SHP-2 (#3752), all of which were obtained from Cell Signaling Technology, as well as with those to *BRAP* (sc-166012), to neurofibromin (sc-376886), and to α-tubulin (sc-32293) from Santa Cruz Biotechnology. Immune complexes were detected with horseradish peroxidase-conjugated secondary antibodies (GE Healthcare and Dako), enhanced chemiluminescence reagents (ImmunoStar LD, Wako), and a LAS-3000mini instrument (GE Healthcare, Chicago, IL, USA). After the detection of the phosphorylated forms of Akt, ERK1/2, MEK, S6, and STAT3, the membrane was stripped with WB Stripping Solution (Nacalai Tesque) and then reprobed with antibodies to the corresponding total forms of these proteins.

### 2.11. Immunofluorescence Analysis

Immunofluorescence analysis was performed with antibodies to phosphorylated MEK (#9129, Cell Signaling Technology, Danvers, MA, USA) and Alexa Fluor 594-conjugated secondary antibodies (#A11037, Invitrogen). Nuclei were stained with Hoechst 33342 (Sigma-Aldrich, St. Louis, MO, USA), and cells were observed with a BZ-9000 microscope (Keyence, Osaka, Japan).

### 2.12. Immunohistochemistry

Immunohistochemical staining was performed as described previously [20] with antibodies to Ki67 (Dako, Glostrup, Denmark) and to S-100 (Dako).

### 2.13. Quantitative RT-PCR Analysis

Reverse transcription (RT) and real-time PCR analysis were performed as previously described [19] with PCR primers (forward and reverse, respectively) as follows: 5′-CGTCCCGTAGACAAAATGGT-3′ and 5′-GAATTTGCCGTGAGTGGAGT-3′ for *GAPDH*; 5′-AGGGGCGATCCAGAACAACG-3′ and 5′-ATGGTCGTAGGGGCTTTCTC-3′ for *BRAP*; and 5′-ACCGCCGTCATTTATCCTGAG-3′ and 5′-CATCTGGTGTTCCGTTTTCATCA-3′ for *PTPN11*.

### 2.14. Statistical Analysis

Data are presented as means ± SD and were analyzed with the two-tailed unpaired Student’s *t* test. A *p* value of <0.05 was considered statistically significant.

## 3. Results

### 3.1. Establishment of Tumor-Derived Cells after sNF96.2-GFP Cell Injection into Nude Mice

**Figure 1:**
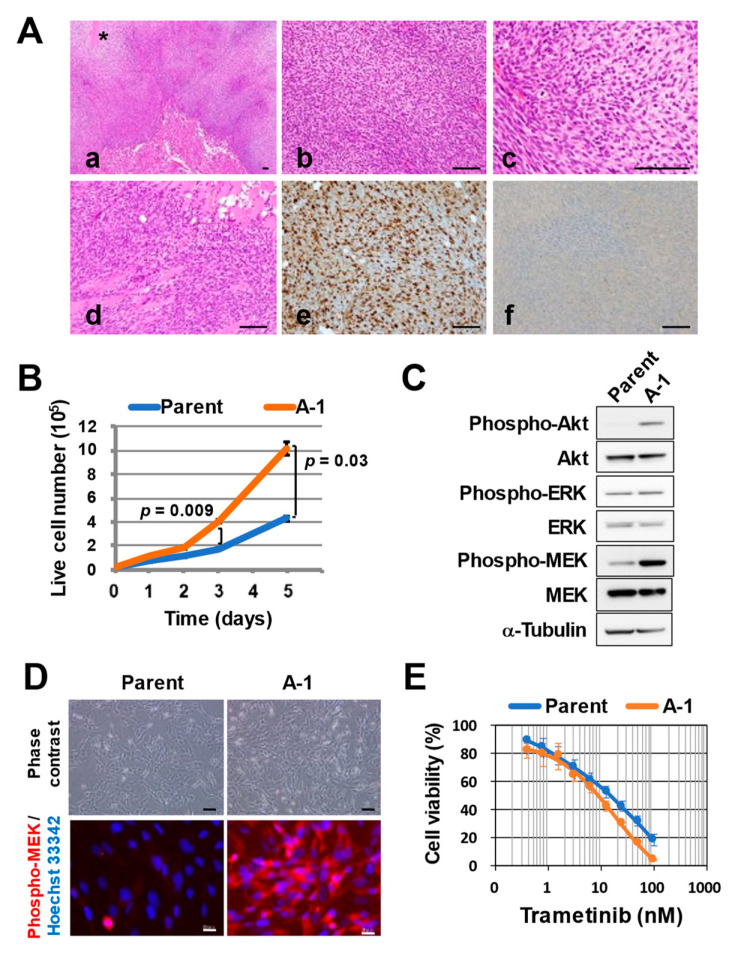
Establishment of tumor-derived cells via sNF96.2-GFP cell injection into a nude mouse. (**A**) Representative hematoxylin–eosin staining (**a**–**d**) and immunohistochemical staining of Ki67 (**e**) or S-100 (**f**) for a tumor formed at 154 days after kidney subcapsular injection of sNF96.2-GFP cells into a Balb/c nu/nu mouse. Scale bars, 100 µm. Invasion of tumor cells into skeletal muscle is shown in image (**d**). The asterisk indicates a necrotic area. (**B**) Comparison of cell proliferative ability in vitro between sNF96.2-GFP (parent) and A-1 cells. Trypan-blue-negative cells were counted at the indicated times. Data are means ± SD from three independent experiments, and the *p* values were calculated with the two-tailed unpaired Student’s *t* test. (**C**) Immunoblot analysis of phosphorylated and total forms of Akt, ERK (extracellular signal-regulated kinase), and MEK (ERK kinase) in sNF96.2-GFP (parent) and A-1 cells. α-Tubulin was examined as a loading control. The uncropped western blot figures are shown in Figure S5. (**D**) Representative phase-contrast microscopy (scale bars, 100 µm) and immunofluorescence staining of phospho-MEK (scale bars, 20 µm) in sNF96.2-GFP (parent) and A-1 cells. Nuclei were stained with Hoechst 33342. (**E**) Comparison of the effects of trametinib on cell viability after incubation of sNF96.2-GFP (parent) or A-1 cells with the MEK inhibitor for 72 h. Data are means ± SD for four replicates of a representative experiment.

As we previously reported, sNF96.2 cells generally show low tumorigenic activity in mice. We found that injecting sNF96.2 cells into the subcutaneous tissues or into the sciatic nerves of immunodeficient mice did not result in tumor formation [19]. We injected a GFP-expressing human *NF1*-MPNST cell line, sNF96.2-GFP, which harbors a frameshift mutation (c.3683delC, p.Asn1229MetfsTer11) in the *NF1* gene, under the kidney capsule of an immunodeficient mouse. The developed tumor at 154 days after cell injection manifested as a high-grade, atypical, spindle-shaped cell sarcoma with necrotic areas (Figure 1(Aa–Ac)) and was diagnosed as spindle cell sarcoma, consistent with an MPNST, on the basis of the histological findings. Many mitotic cells were observed in the tumor, with a Ki-67 labeling rate of >60%, indicating that it consisted of highly proliferative tumor cells (Figure 1(Ae)). The tumor cells had spread outside of the kidney and infiltrated skeletal muscle (Figure 1(Ad)). We performed immunostaining for S-100 (Figure 1(Af)) as well as for Sox10, glial fibrillary acidic protein (GFAP), and CD57 (data not shown), all of which are commonly examined for the diagnosis of human MPNST. About half of all human MPNST cases are negative for these proteins, and the developed tumor was also negative.

We then established cells from the developed tumor and designated them as A-1 cells. To eliminate the possibility of cell contamination, we performed STR analysis. The STR profile of the A-1 cells was identical to that of the parental sNF96.2-GFP cells, and we confirmed that these latter cells were the same as those registered with ATCC (CRL-2884 sNF96.2) by comparison with the JCRB Cell Bank database (Figure S1). We found that the proliferative activity of A-1 cells in vitro was significantly higher than that of the parental sNF96.2-GFP cells (Figure 1B). Immunoblot analysis and immunofluorescence analysis also revealed that the phosphorylation (activation) levels of Akt and MEK were much higher in A-1 cells than in sNF96.2-GFP cells (Figure 1C,D). In addition, A-1 cells were more sensitive to the MEK inhibitor trametinib than sNF96.2-GFP cells were (Figure 1E).

### 3.2. Identification of Genetic Alterations Related to *NF1* Tumor Malignancy

**Figure 2:**
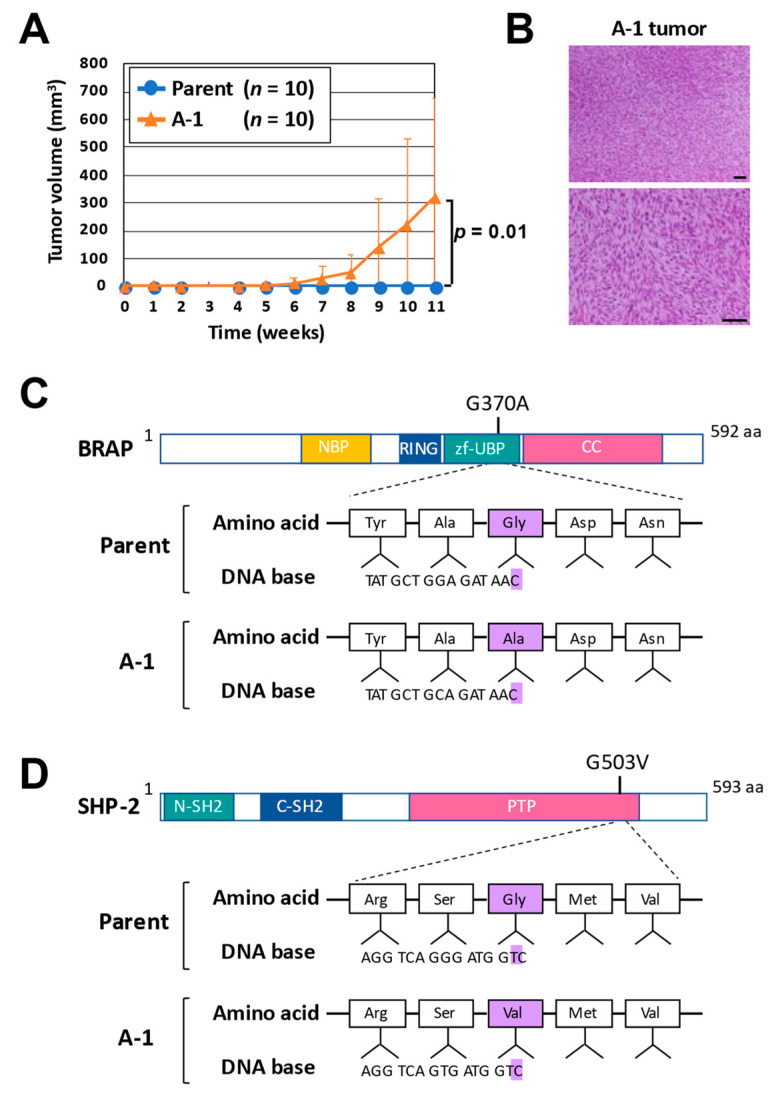
Identification of genetic alterations related to *NF1* tumor malignancy. (**A**) Tumor volume measured at the indicated times after subcutaneous injection of sNF96.2-GFP (parent) or A-1 cells (5 × 10^6^) into Balb/c nu/nu mice. Data are means ± SD (*n* = 10 mice), and the *p* value was calculated with the two-tailed unpaired Student’s *t* test. (**B**) Representative hematoxylin–eosin staining of a tumor formed at 11 weeks after subcutaneous injection of A-1 cells. Scale bars, 100 µm. (**C**) Domain organization of human *BRAP* and location of the amino acid difference between sNF96.2-GFP (parent) and A-1 cells. *BRAP* encodes BRCA1-associated protein (*BRAP*, also known as RNF52, *BRAP*2, and IMP), which contains a nucleotide-binding a/b plait (NBP) domain, a really interesting new gene zinc finger (RING) domain, a ubiquitin-specific protease (UBP)-like zinc finger (zf-UBP) domain, and a coiled-coil (CC) domain and functions as a *RAS*-responsive E3 ubiquitin ligase [21]. (**D**) Domain organization of human SHP-2 and location of the amino acid difference between sNF96.2-GFP (parent) and A-1 cells. *PTPN11* encodes protein tyrosine phosphatase nonreceptor type 11 (SHP-2), which contains an NH_2_-terminal SRC homology 2 (N-SH2) domain, a COOH-terminal SRC homology 2 (C-SH2) domain, and a protein tyrosine phosphatase (PTP) domain [22].

We also found that the tumorigenic activity of A-1 cells was much higher than that of the parental sNF96.2-GFP cells when the cells were injected subcutaneously into nude mice (Figure 2A). The tumors formed after the subcutaneous injection of A-1 cells recapitulated the histological features of those formed by sNF96.2-GFP cells in the kidney (Figure 2B). To identify additional genetic alterations that promote the malignancy of *NF1*-associated tumors, we analyzed the genomic DNA of sNF96.2-GFP parental cells and four subclones of tumor-derived cells (Figure S2). Three additional cell subclones (B-1, C-1, and D-1) were thus established from tumors formed after the injection of sNF96.2-GFP cells below the renal capsule of immunodeficient mice. Next-generation sequencing of the genomic DNA was performed in order to compare the whole-exome sequences of the parental cells and four subclones (A-1, B-1, C-1, and D-1). The *NF1* germline mutation was identified as a biallelic base pair deletion in exon 21 (3683delC) that results in a frameshift and the generation of a premature stop codon before the *RAS*-GAP domain of the encoded protein. In addition, the tumor-derived cells showed the somatic loss of heterozygosity on chromosome 17. The MuTect program revealed that, whereas each of the four subclones harbored unique genetic alterations, all four subclones shared mutations in 42 genes that were not present in the parental cells (Figure S3). Among these 42 genes, 13 genes are related to cancer and other diseases (Table 1). Of note, three subclones established from secondary tumors formed after A-1 cell injection (Figure S2) did not harbor any common alterations other than these 13 gene variants. We therefore speculated that these 13 gene variants might be associated with the malignant progression of *NF1*-associated tumors. Although the *NF1* gene encodes a *RAS*-GAP, *NF1* mutation appears to not be sufficient to induce the persistent activation of the *RAS*-ERK pathway. Given that the phosphorylation levels of Akt and MEK were increased in the tumor-derived A-1 cells, we focused on the relation of additional mutations that might affect this pathway to tumor malignancy.

Among these 13 genes, we have a special interest in the *BRAP* and *PTPN11* mutations because these are the regulators that control the activity of the *RAS*-ERK pathway. We identified missense mutations of *BRAP* (c.1109G > C, p.Gly370Ala) and *PTPN11* (c.1508G > T, p.Gly503Val) in the tumor-derived subclones but not in the parental sNF96.2-GFP cells (Figure 2C,D; Figure S4A,B). Quantitative RT-PCR analysis showed that the abundance of *BRAP* and *PTPN11* mRNAs did not differ substantially between sNF96.2-GFP and A-1 cells (Figure S4C,D).

### 3.3. Role of Missense Mutations of BRAP and PTPN11 in Tumorigenic Activity

**Figure 3:**
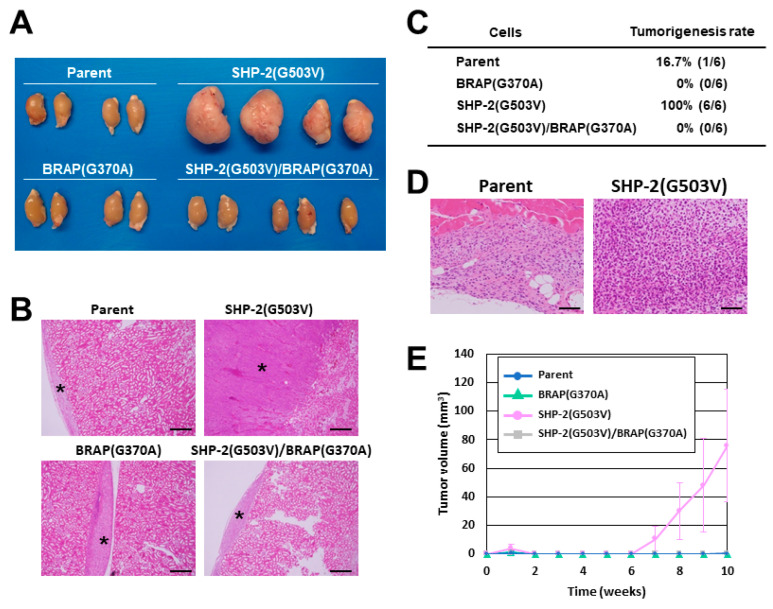
Tumor formation by sNF96.2 cells harboring missense mutations of *BRAP* and *PTPN11*. (**A**) Macroscopic appearance of the kidneys of Balb/c nu/nu mice at 3 months after renal subcapsular injection of sNF96.2-GFP (Parent) or SHP-2 (G503V)-, *BRAP* (G370A)-, or SHP-2 (G503V)/*BRAP* (G370A)-expressing sNF96.2 cells (1 × 10^6^). (**B**) Representative hematoxylin–eosin staining of tumors in (**A**). Asterisks indicate tumor tissue. Scale bars, 500 µm. (**C**) Tumorigenic rate for the indicated cells (5 × 10^6^) at 70 days after subcutaneous injection in Balb/c nu/nu mice. (**D**) Representative hematoxylin–eosin staining of tumors in **C**. Scale bars, 100 µm. (**E**) Time course of tumor volume for tumors in **C**. Data are means ± SD (*n* = 6).

To determine the role of these mutations of *BRAP* and *PTPN11* in the malignant progression of *NF1*-associated tumors, we established three sNF96.2 cell lines that stably overexpress *BRAP* (G370A), SHP-2 (G503V), or both *BRAP* (G370A) and SHP-2 (G503V). We injected these cells as well as the parental sNF96.2-GFP cells either below the kidney capsule or subcutaneously in nude mice. The tumors formed below the kidney capsule by the SHP-2 (G503V)-expressing cells were much larger than those formed by the parental cells (Figure 3A,B). Only parental and SHP-2 (G503V)-expressing cells formed subcutaneous tumors, with formation rates of 16.7% (1/6) and 100% (6/6), respectively (Figure 3C). The subcutaneous tumors formed by SHP-2 (G503V)-expressing cells manifested a higher density of tumor cells (Figure 3D) and were larger (Figure 3E) compared with those formed by the parental cells, with the mean tumor volume at 10 weeks after cell injection being 75.89 versus 1.69 mm^3^. The higher tumorigenic activity of SHP-2 (G503V)-expressing cells relative to parental sNF96.2-GFP cells after both kidney subcapsular and subcutaneous injection thus suggested that additional mutation of *PTPN11* is able to enhance the malignant characteristics of *NF1*-associated tumors. In contrast, cells expressing *BRAP* (G370A) or both *BRAP* (G370A) and SHP-2 (G503V) did not form tumors in subcutaneous tissue (Figure 3C,E), and the tumors formed below the kidney capsule by these cells were of a similar size to those formed by parental cells (Figure 3A,B), suggesting that *BRAP* (G370A) suppresses SHP-2 (G503V)-mediated tumorigenic activity.

### 3.4. Effects of Missense Mutations in BRAP and PTPN11 on MEK Phosphorylation

**Figure 4:**
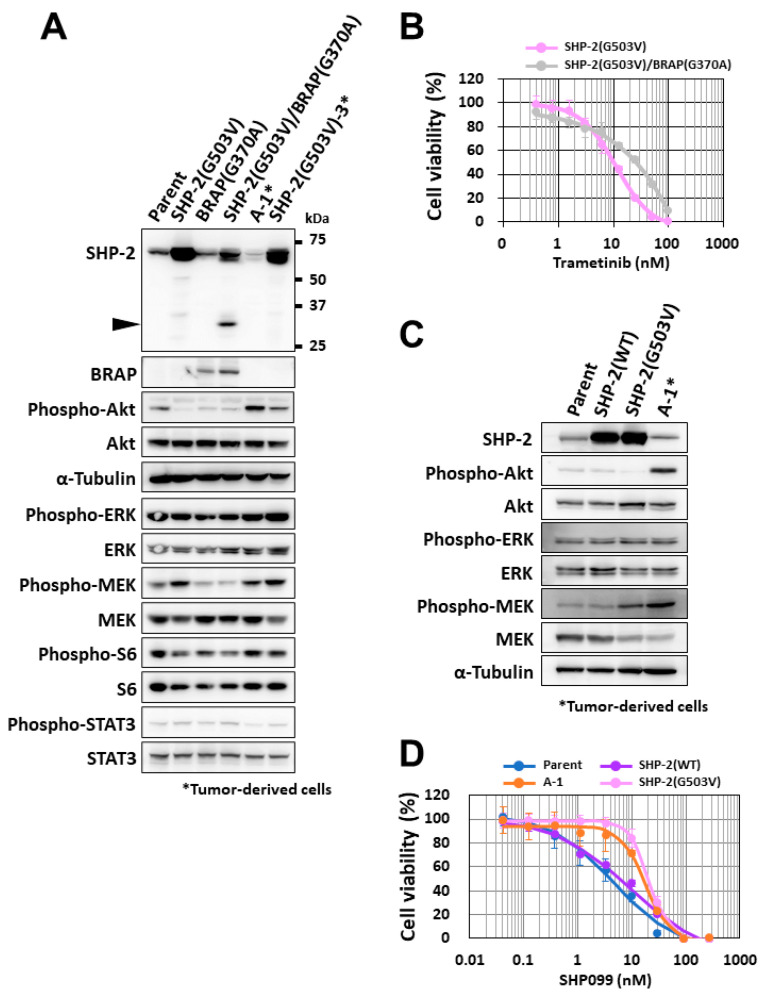
Role of missense mutations of *BRAP* and *PTPN11* in tumor malignancy. (**A**) Immunoblot analysis of SHP-2, *BRAP*, and both phosphorylated and total forms of Akt, ERK, MEK, S6, and STAT3 (signal transducer and activator of transcription 3) in sNF96.2-GFP (parent) cells, A-1 cells, SHP-2 (G503V)-expressing cells, *BRAP* (G370A)-expressing cells, SHP-2 (G503V)/*BRAP* (G370A)-expressing cells, and cells derived from a kidney subcapsular tumor formed by SHP-2 (G503V)-expressing cells (SHP-2 (G503V)-3 cells). Arrowhead indicates a lower-molecular-weight protein reactive with the antibodies to SHP-2. The uncropped western blot figures are shown in Figure S5. (**B**) Comparison of the effects of trametinib on cell viability after incubation of SHP-2 (G503V)-expressing and SHP-2 (G503V)/*BRAP* (G370A)-expressing cells with the MEK inhibitor for 72 h. Data are means ± SD for four replicates of a representative experiment. (**C**) Immunoblot analysis of SHP-2 as well as of phosphorylated and total forms of Akt, ERK, and MEK in sNF96.2-GFP (parent) cells, A-1 cells, SHP-2(WT)-expressing cells, and SHP-2 (G503V)-expressing cells. The uncropped western blot figures are shown in Figure S5. (**D**) Comparison of the effects of SHP099 on cell viability after incubation of cells as in (**C**) with the SHP-2 inhibitor for 72 h. Data are means ± SD for four replicates of a representative experiment.

Immunoblot analysis revealed that, as in A-1 cells, the phosphorylation level of MEK was increased in SHP-2 (G503V)-expressing cells as well as in cells derived from a tumor formed by these cells (SHP-2 (G503V)-3 cells), whereas MEK phosphorylation in *BRAP* (G370A)- or SHP-2 (G503V)/*BRAP* (G370A)-expressing cells did not differ from that in parental cells (Figure 4A). The phosphorylation level of Akt was also increased in the SHP-2 (G503V) tumor-derived cells, as it was in A-1 cells (Figure 4A). Consistent with these results, SHP-2 (G503V)-expressing cells showed a greater sensitivity to trametinib than SHP-2 (G503V)/*BRAP* (G370A)-expressing cells did (Figure 4B). We next established sNF96.2 cells that overexpress wild-type (WT) SHP-2 and compared them with those expressing SHP-2 (G503V). Immunoblot analysis revealed that, whereas MEK phosphorylation was increased in SHP-2 (G503V)-expressing cells, it was unaffected in the SHP-2 (WT)-expressing cells (Figure 4C). We also performed a cytotoxicity assay with the SHP-2 inhibitor SHP099 [23] and found that the sensitivity of SHP-2 (WT)-expressing cells to this agent was similar to that of parental cells, whereas SHP-2 (G503V)-expressing cells as well as tumor-derived A-1 cells were less sensitive to SHP099 (Figure 4D).

### 3.5. Mechanism of MEK Inhibition by BRAP Missense Mutation

**Figure 5:**
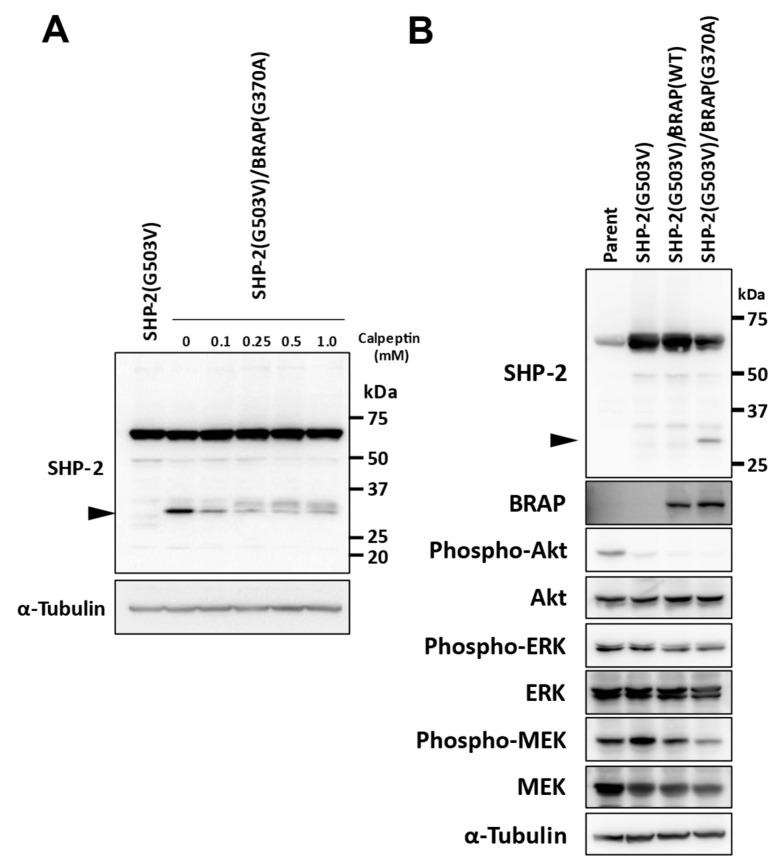
Mechanism of MEK inhibition by *BRAP* missense mutation. (**A**) Immunoblot analysis of SHP-2 in SHP-2 (G503V)-expressing sNF96.2-GFP cells as well as in SHP-2 (G503V)/*BRAP* (G370A)-expressing sNF96.2-GFP cells incubated in the absence or presence of calpeptin (0.1 to 1 mM) for 1 h. The uncropped western blot figures are shown in Figure S5. (**B**) Immunoblot analysis of SHP-2, *BRAP*, as well as phosphorylated and total forms of Akt, ERK, and MEK in sNF96.2-GFP (parent) cells, SHP-2 (G503V)-expressing cells, SHP-2 (G503V)/*BRAP* (WT)-expressing cells, and SHP-2 (G503V)/*BRAP* (G370A)-expressing cells. Arrowheads indicate a lower-molecular-weight protein reactive with the antibodies to SHP-2. The uncropped western blot figures are shown in Figure S5.

The identification of the mechanism by which *BRAP* (G370A) suppresses SHP-2 (G503V)-mediated tumorigenic activity may inform the development of new therapeutic approaches to the inhibition of the *RAS*-ERK pathway. Given that a lower-molecular-weight protein reactive with antibodies to SHP-2 was detected when *BRAP* (G370A) was coexpressed with SHP-2 (G503V) (Figure 4A), we considered the possibility that SHP-2 (G503V) might undergo proteolytic cleavage in a manner dependent on *BRAP* (G370A). SHP-2 has previously been shown to be cleaved in a calpain-dependent manner [24], and we found that the calpain inhibitor calpeptin attenuated the generation of the immunoreactive lower-molecular-weight protein in a concentration-dependent manner in cells expressing both SHP-2 (G503V) and *BRAP* (G370A) (Figure 5A).

We next established sNF96.2 cells that stably express the WT form of *BRAP* in order to clarify the relevance of the G370A variant. Immunoblot analysis revealed that both the cleavage of SHP-2 and inhibition of MEK phosphorylation apparent in SHP-2 (G503V)-expressing cells that also express *BRAP* (G370A) were much less pronounced in those expressing *BRAP* (WT) (Figure 5B). These data suggested that the mutant form of *BRAP* mediates SHP-2 (G503V) cleavage and thereby downregulates MEK phosphorylation.

### 3.6. Relation of Phenotypes Induced by BRAP and PTPN11 Missense Mutations to NF1 Loss

**Figure 6:**
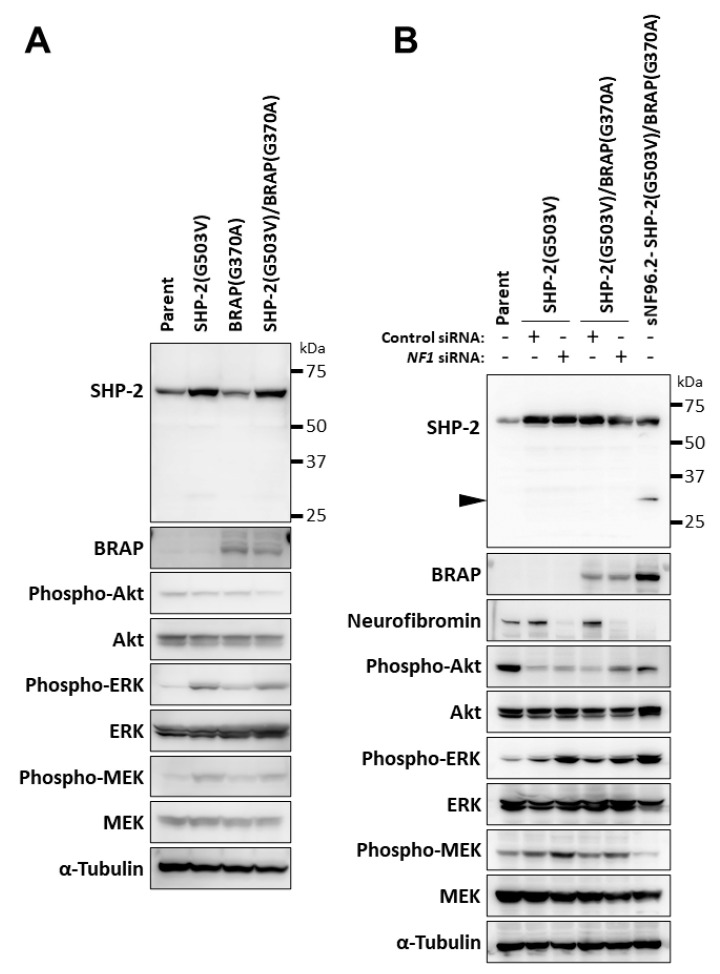
Relation of phenotypes induced by *BRAP* and *PTPN11* missense mutations to *NF1* inactivation. Immunoblot analysis was performed for SHP-2, *BRAP*, neurofibromin, as well as phosphorylated and total forms of Akt, ERK, and MEK in parental HeLa cells as well as HeLa cells transiently expressing SHP-2 (G503V) or *BRAP* (G370A). The uncropped western blot figures are shown in Figure S5. (**A**) or in the corresponding stably infected cells transfected with control or *NF1* siRNAs for 48 h (**B**). sNF96.2-GFP cells expressing SHP-2 (G503V) and *BRAP* (G370A) are shown for comparison to indicate the cleavage product of SHP-2 (arrowhead). The uncropped western blot figures are shown in Figure S5.

To determine whether the inactivation of *NF1* is required for the effects of SHP-2 (G503V) and *BRAP* (G370A) on MEK activation, we transiently expressed SHP-2 (G503V) and *BRAP* (370A) in HeLa cells, which are WT for *NF1*. The expression of SHP-2 (G503V) resulted in a small increase in MEK phosphorylation and a more marked increase in ERK phosphorylation in HeLa cells (Figure 6A). Whereas the coexpression of *BRAP* (G370A) with SHP-2 (G503V) attenuated MEK phosphorylation and induced the cleavage of SHP-2 (G503V) in sNF96.2 cells (Figure 4A), these changes were not observed in HeLa cells (Figure 6A). Of note, ERK activation induced by SHP-2 (G503V) was not suppressed by *BRAP* (G370A) in HeLa cells (Figure 6A).

Finally, we examined the effects of the knockdown of neurofibromin by siRNA transfection in HeLa cells stably expressing SHP-2 (G503V) or both SHP-2 (G503V) and *BRAP* (G370A) (Figure 6B). In the SHP-2 (G503V)-expressing cells, the knockdown of neurofibromin increased the phosphorylation of MEK and ERK, but not that of Akt, indicating that MEK-ERK signaling was preferentially activated. The activation of MEK by SHP-2 (G503V) was thus also confirmed in the neurofibromin-depleted HeLa cells. The knockdown of neurofibromin tended to increase the phosphorylation of MEK and ERK in HeLa cells expressing both *BRAP* (G370A) and SHP-2 (G503V). The comparison of neurofibromin-depleted SHP-2 (G503V)-expressing cells and neurofibromin-depleted SHP-2 (G503V)/*BRAP* (G370A)-expressing cells revealed that the phosphorylation of Akt was increased and that of MEK and ERK was slightly decreased in the latter. The cleavage of SHP-2 was not observed in HeLa cells, however.

## 4. Discussion

Genomic DNA sequencing allowed us to identify additional genetic alterations including mutations in the *PTPN11* (encoding SHP-2) and *BRAP* genes that were associated with enhanced *NF1*-related tumor malignancy. *BRAP* negatively regulates ERK activation by inactivating the KSR1 scaffold protein and limiting the formation of the RAF-MEK complex [25,26,27]. Upon the activation of *RAS*, *BRAP* binds to *RAS*-GTP and undergoes autoubiquitylation, resulting in the stabilization of the KSR1-RAF-MEK complex and the activation of MEK-ERK signaling [26]. SHP-2 is a member of the PTP family and regulates SRC family kinase activity and *RAS*-ERK activation [28]. We found that the forced expression of the mutant form of *PTPN11* (c.1508G > T, p.Gly503Val) promoted MEK activation and tumorigenic activity in neurofibromin-deficient cells, whereas these effects were attenuated by the coexpression of the mutant form of *BRAP* (c.1109G > C, p.Gly370Ala). Moreover, the suppressive effect of the *BRAP* (G370A) protein on MEK phosphorylation was not observed in neurofibromin-intact cells. Our data indicate that *PTPN11* mutation drives malignancy in *NF1*-mutated cells and that the inhibition of SHP-2 by *BRAP* may provide the basis for a new therapeutic strategy for malignant *NF1*-associated tumors.

Evidence from transgenic mouse models has implicated dysfunction of the proteins p53, Rb, and p16 in the malignant progression of *NF1*-associated tumors [29]. In addition, somatic mutations of *SUZ12* and *EED* which encode core components of the Polycomb repressive complex 2 (PRC2) have been frequently identified in human *NF1*-associated MPNSTs, indicating that mutations of these genes play a role in the establishment of these tumors [30,31,32]. Our whole-exome sequence analysis revealed that the parental sNF96.2-GFP cells examined in this study harbor a homozygous missense mutation in *EED* (p.S241R). Zebrafish models have also shown that the overexpression of *PDGFRA* cooperates with the loss of *NF1* and the p53 gene to promote MPNST formation [33]. Furthermore, the activation of the PI3K (phosphatidylinositol 3-kinase)–Akt–mTOR (mammalian target of rapamycin) signaling pathway caused by the deletion of the *PTEN* gene has been shown to induce the malignant transformation of *NF1*-associated tumors [34]. We found that the phosphorylation of Akt was increased in tumor-derived sNF96.2 cells (A-1 cells), suggesting that Akt was activated in all tumor cells or that tumor cells with activated Akt were selected in vivo. We also found that the phosphorylation level of MEK was increased in tumor-derived cells as well as in sNF96.2 cells stably expressing SHP-2 (G503V). Both the *RAS*-ERK and PI3K-Akt-mTOR signaling pathways thus appear to play key roles in the malignant transformation of neurofibroma into MPNST.

MEK-ERK signaling contributes to the regulation of cellular functions such as proliferation, differentiation, and apoptosis as well as to that of normal tissue and tumor formation [35]. Molecular and pharmacological studies have shown that MEK and ERK are required for the transforming activities of *RAS* and various other oncogenes, and preclinical data support an important role for the *RAS*-ERK pathway in cancer biology and its potential as a therapeutic target in human cancers [36]. The inhibition of MEK-ERK signaling was found to restore colon tumor cells to a nontransformed state in vitro and to inhibit tumor growth in vivo [37], suggesting that increased activity of such signaling is related to tumor development. We found that ERK was constitutively phosphorylated and that the expression of SHP-2 (G503V) increased the level of MEK phosphorylation in *NF1*-mutated cells, indicating that the activity of the MEK-ERK pathway was increased further by the pathogenic mutation of *PTPN11* in these cells. Such enhanced activation of MEK-ERK signaling may promote the transformation of benign neurofibroma cells into malignant tumor cells.

The *PTPN11* missense mutation identified in the present study induced pathological MEK activation and promoted tumor malignancy. Constitutional gain-of-function mutations of *PTPN11* cause Noonan syndrome in humans as a result of the activation of *RAS* signaling [38]. *PTPN11* mutations have been detected in human cancers including leukemias and solid tumors, with the G503V missense mutation being associated with childhood leukemia [39] and adult acute myelogenous leukemia [40]. Although the 13 genes that we identified were not on the list of genes identified using a forward genetic screen in Schwann cells utilizing the *Sleeping Beauty* (*SB*) transposon-based somatic mutagenesis system in mice to determine the driver genes of MPNST [41], *PTPN11* missense mutation and *NF1* homozygous loss were recently detected in clinical specimens of MPNSTs [42], suggesting that the combination of *PTPN11* and *NF1* mutations may give rise to the persistent activation of both *RAS*-ERK and PI3K-Akt-mTOR pathways and to malignant transformation. A small-molecule allosteric inhibitor of SHP-2 (RMC-4550) was found to inhibit the activity of oncogenic *RAS*-ERK signaling and cancer growth in models of human cancer harboring mutated *BRAF*, *NF1* loss, or the G12C mutation of *KRAS* [43], suggesting that the inhibition of SHP-2 is a promising molecular therapeutic strategy for *NF1*-associated tumors. The combination of a MEK inhibitor plus the SHP-2 inhibitor SHP099 was recently shown to be effective in models of *NF1*-MPNST and plexiform neurofibroma [44]. However, our data show that SHP-2 (G503V)-expressing sNF96.2 cells were less sensitive to SHP099 than cells overexpressing SHP-2 (WT) were.

We found that the expression of *BRAP* (G370A), but not that of *BRAP* (WT), markedly attenuated MEK phosphorylation in sNF96.2 cells expressing SHP-2 (G503V) and that *BRAP* (G370A) suppressed SHP-2 (G503V)-mediated tumorigenic activity in these cells. We detected a lower-molecular-weight protein derived from SHP-2 in cells coexpressing *BRAP* (G370A) and SHP-2 (G503V), suggesting the possibility of proteolytic cleavage of SHP-2 (G503V) dependent on *BRAP* (G370A). SHP-2 was previously shown to undergo calpain-dependent cleavage during cell death induced by *Entamoeba histolytica* infection in Jurkat T cells [24]. We found that the calpain inhibitor calpeptin blocked the generation of the SHP-2 cleavage product in sNF96.2 cells coexpressing SHP-2 (G503V) and *BRAP* (G370A), implicating calpain in SHP-2 (G503V) cleavage. The function of SHP-2 (G503V) might be expected to be suppressed by such cleavage. *BRAP* is an E3 ubiquitin ligase and possesses RING and zf-UBP domains [21], suggesting that SHP-2 may be a direct target of *BRAP*. The precise mechanism underlying the cleavage of SHP-2 (G503V) in malignant tumor cells requires further study.

We identified 14 heterozygous mutations within the coding regions of 13 genes—*AMER1*, *BCAT1*, *BRAP*, *C3*, *CNTNAP2*, *COL6A6*, *NOS3*, *PCNT*, *PTPN11*, *SCNN1B*, *SLC5A11*, *SYNE1*, and *WIPF1*—that were commonly present in highly malignant *NF1*-mutated cells. We speculate that the cell subclones with mutations taking advantages for proliferation and survival in vivo were selected and grew in mice. Given that the phosphorylation of Akt and MEK was increased in the malignant A-1 cells, among these 13 genes, we focused on those (*BRAP* and *PTPN11*) related to the *RAS*-ERK pathway. Our results suggest that the *BRAP* mutation might have developed as a negative feedback response to the *PTPN11* mutation. Whereas the overexpression of *BRAP* (G370A) strongly suppressed MEK activity mediated by the overexpression of SHP-2 (G503V), in A-1 cells, *BRAP* (G370A) may adjust MEK activity so as to stabilize cell survival and proliferation, suggesting that the expression levels may be related to the suppressive effect of mutant *BRAP*. The *BRAP* mutation may thus serve to modulate cell survival and proliferation signals in order to optimize the growth of malignant *NF1*-mutated cells. Further characterization of the other gene alterations found to be associated with malignant transformation in our study may provide the basis for a better understanding of *NF1*-MPNST.

The cleavage of SHP-2 (G503V) and suppression of MEK phosphorylation mediated by *BRAP* (G370A) were apparent in *NF1*-mutated (sNF96.2) cells but not in *NF1*-intact (HeLa) cells, suggesting that the phenotypes induced by *BRAP* and *PTPN11* missense mutations are related to *NF1* loss. Whereas the marked activation of MEK by SHP-2 (G503V) was detected in neurofibromin-depleted HeLa cells, the cleavage of SHP-2 was not, suggesting that the attenuation of MEK phosphorylation and cleavage of SHP-2 (G503V) dependent on *BRAP* (G370A) may only occur in cells in which *NF1* is chronically inactivated. Different downstream signals may be activated depending on whether *NF1* expression is acutely or chronically deficient. Indeed, sNF96.2 cells showed a high level of ERK phosphorylation, whereas neurofibromin depletion increased ERK phosphorylation without affecting Akt phosphorylation in SHP-2 (G503V)-expressing HeLa cells. Furthermore, the knockdown of neurofibromin tended to increase the phosphorylation of MEK and ERK in SHP-2 (G503V)/*BRAP* (G370A)-expressing HeLa cells. Our findings indicate that tumor progression induced by SHP-2 (G503V) and its suppression by *BRAP* (G370A) may provide the basis for the development of new strategies for the treatment of *NF1*-MPNST as well as for the prevention of the malignant transformation of neurofibroma in individuals with *NF1*.

## 5. Conclusions

Here, we showed that MEK phosphorylation was increased in a highly malignant subclone of *NF1*-MPNST cells. Genomic DNA sequencing identified missense mutations of *PTPN11* (encoding SHP-2) and *BRAP* in these cells. We also found that the forced expression of the SHP-2 mutant promoted MEK activation and malignancy in the parental *NF1*-MPNST cells, and that these effects of the SHP-2 mutant were attenuated by the forced expression of the *BRAP* mutant. The combination of *PTPN11* and *NF1* mutations may thus give rise to malignant transformation in *NF1* cells, and SHP-2 inhibition by *BRAP* is a potential therapeutic strategy for *NF1*-MPNST.

## Figures and Tables

**Table 1:** Identification of 14 heterozygous mutations of 13 cancer- and other disease-related genes commonly present in tumor-derived subclones (A-1, B-1, C-1, and D-1). Chrom., chromosome.

Gene Name	Chrom.	Transcript ID: Codon Change	Protein ID: Amino Acid Change
*AMER1*	X	NM_152424.3:c.3382G > T	NP_689637.3:p.Ala1128Ser
*BCAT1*	12	NM_001178093.1:c.600C > G	NP_001171564.1:p.Ser200Arg
*BRAP*	12	NM_006768.3:c.1109G > C	NP_006759.3:p.Gly370Ala
*C3*	19	NM_000064.2:c.4516T > C	NP_000055.2:p.Cys1506Arg
*CNTNAP2*	7	NM_014141.5:c.1573C > A	NP_054860.1:p.Gln525Lys
*COL6A6*	3	NM_001102608.1:c.270G > C	NP_001096078.1:p.Met90Ile
*NOS3*	7	NM_000603.4:c.2260C > A	NP_000594.2:p.His754Asn
*PCNT*	21	NM_006031.5:c.445A > G; c.481G > A	NP_006022.3:p.Ser149Gly; p.Val161Ile
*PTPN11*	12	NM_002834.3:c.1508G > T	NP_002825.3:p.Gly503Val
*SCNN1B*	16	NM_000336.2:c.1023G > C	NP_000327.2:p.Glu341Asp
*SLC5A11*	16	NM_052944.3:c.439G > T	NP_443176.2:p.Val147Leu
*SYNE1*	6	NM_182961.3:c.19303A > G	NP_892006.3:p.Lys6435Glu
*WIPF1*	2	NM_003387.4:c.613C > G	NP_003378.3:p.Pro205Ala

## Data Availability

Representative data are provided in this published article and its Supplementary Material Files. Other data sets generated or analyzed during the current study are available from the corresponding author upon reasonable request.

## Supplementary Materials

The following supporting information can be downloaded at: https://www.mdpi.com/article/10.3390/cancers14102377/s1. Figure S1: Confirmation of the identity of A-1 cells with parental sNF96.2-GFP cells via STR analysis. Figure S2: Experimental scheme for genome DNA analysis of tumor-derived cells. sNF96.2-GFP cells were injected below the renal capsule of immunodeficient mice, and tumor-derived cells were established from each mouse. Figure S3: Identification of 42 genes commonly harboring mutations in tumor-derived cells (A-1, B-1, C-1, and D-1 cells). Figure S4: Alignment of the nucleotide sequences of *BRAP* (A) and *PTPN11* (B) for sNF96.2-GFP (P-G) and A-1 cells as performed with ClustalW (base differences are boxed in red) as well as quantitative RT-PCR analysis of *BRAP* (C) and *PTPN11* (D) mRNA abundance in these cells (data are means ± SD from three independent experiments). Figure S5: The uncropped western blot figures.
