# Supplementary material for: Mutation of PTPN11 (Encoding SHP-2) Promotes MEK Activation and Malignant Progression in Neurofibromin-Deficient Cells in a Manner Sensitive to BRAP Mutation
Source: Cancers (Basel). 2022 May 12;14(10):2377. doi: 10.3390/cancers14102377 (PMC9140047; doi:10.3390/cancers14102377)
Supplement: Supplementary file 1 [file cancers-14-02377-s001.zip › Figure S5.pptx]

## Slide 1
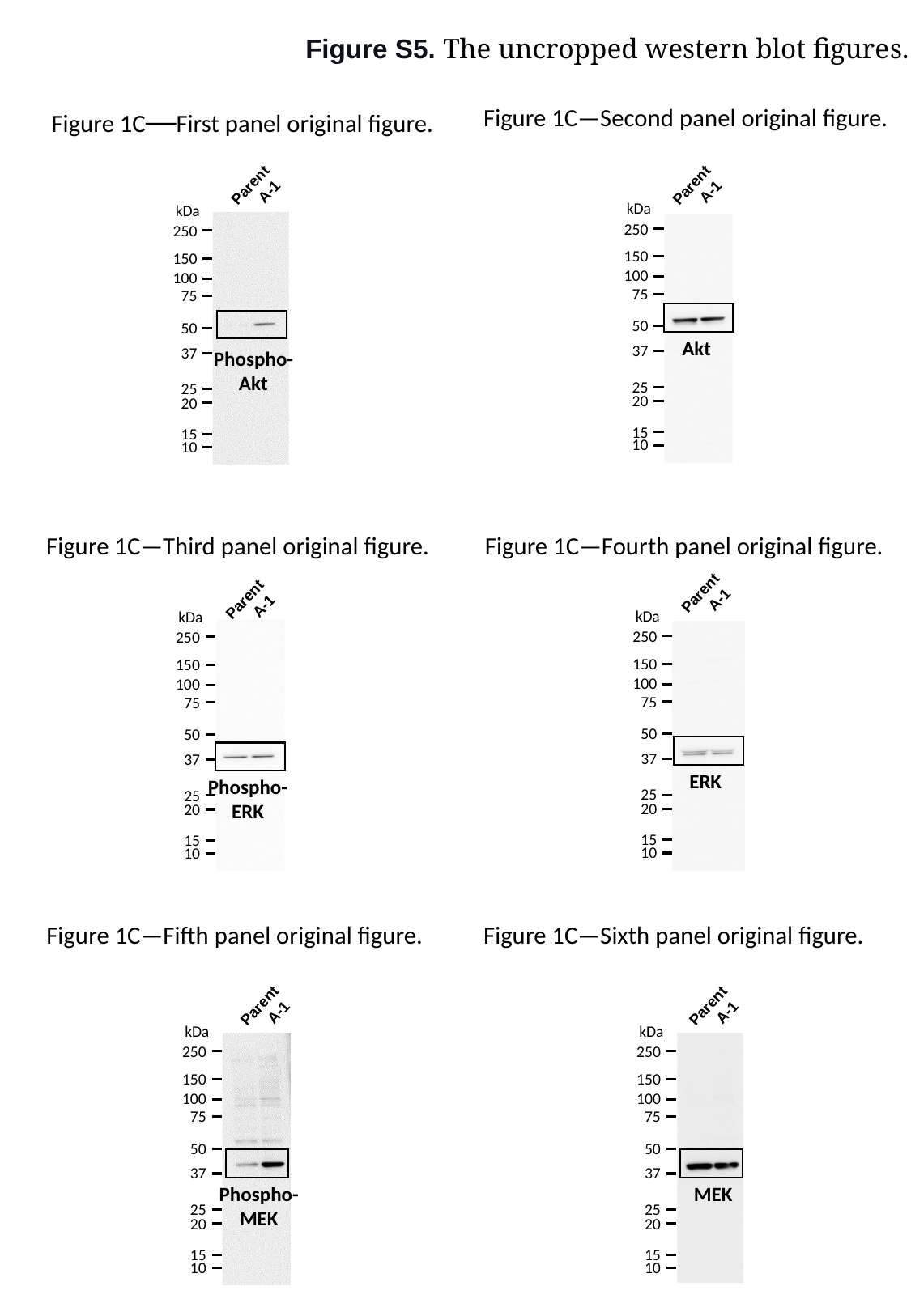

Figure S5. The uncropped western blot figures.
Figure 1C—First panel original figure.
Figure 1C—Second panel original figure.
Parent
A-1
kDa
250
150
100
75
50
37
25
20
15
10
Phospho-
Akt
Parent
A-1
kDa
250
150
100
75
50
37
25
20
15
10
Akt
Figure 1C—Third panel original figure.
Figure 1C—Fourth panel original figure.
Parent
A-1
kDa
250
150
100
75
50
37
25
20
15
10
ERK
Parent
A-1
kDa
250
150
100
75
50
37
25
20
15
10
Phospho-
ERK
Figure 1C—Fifth panel original figure.
Figure 1C—Sixth panel original figure.
Parent
A-1
kDa
250
150
100
75
50
37
25
20
15
10
Phospho-
MEK
Parent
A-1
kDa
250
150
100
75
50
37
25
20
15
10
MEK

## Slide 2
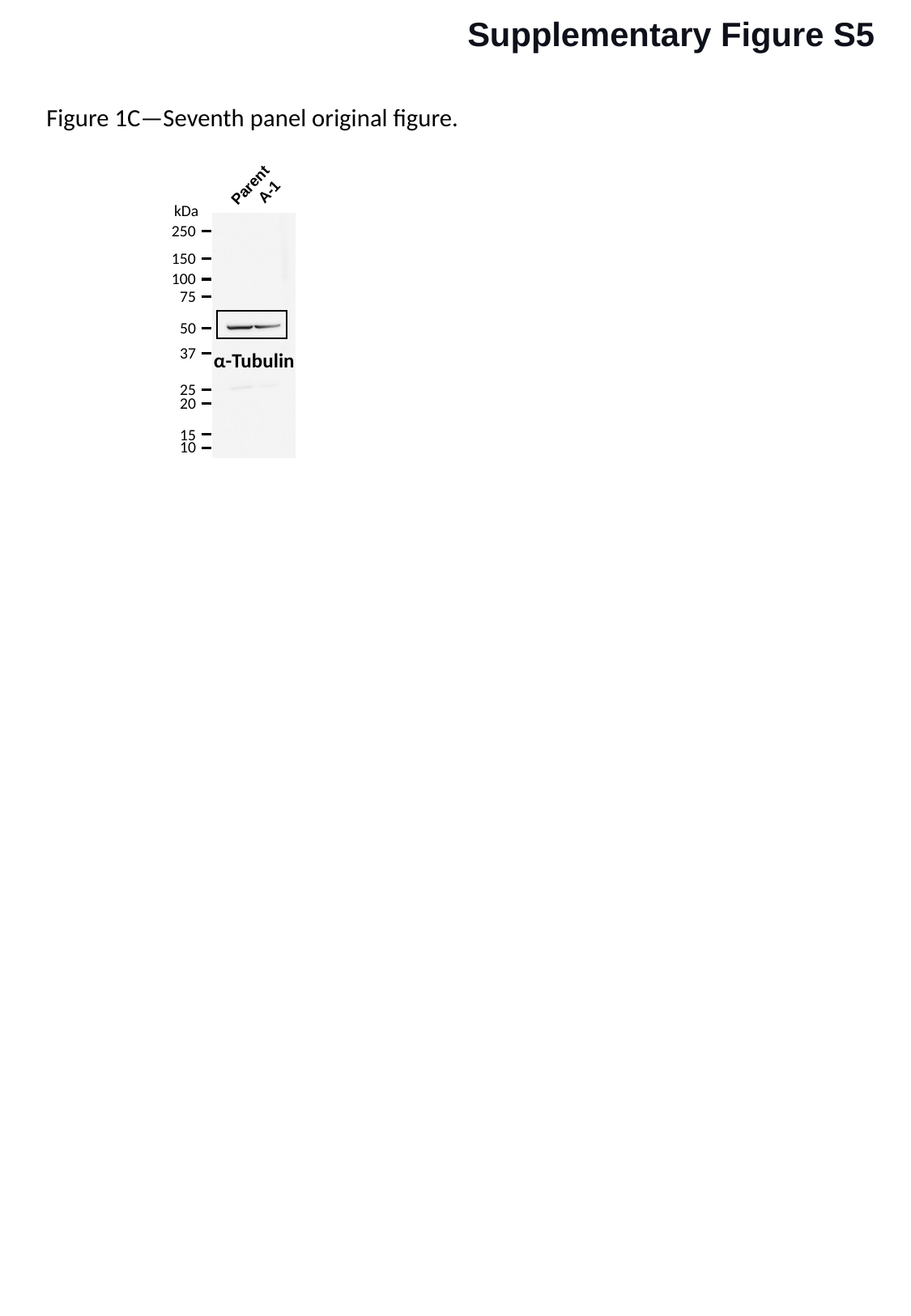

Supplementary Figure S5
Figure 1C—Seventh panel original figure.
Parent
A-1
kDa
250
150
100
75
50
37
25
20
15
10
α-Tubulin

## Slide 3
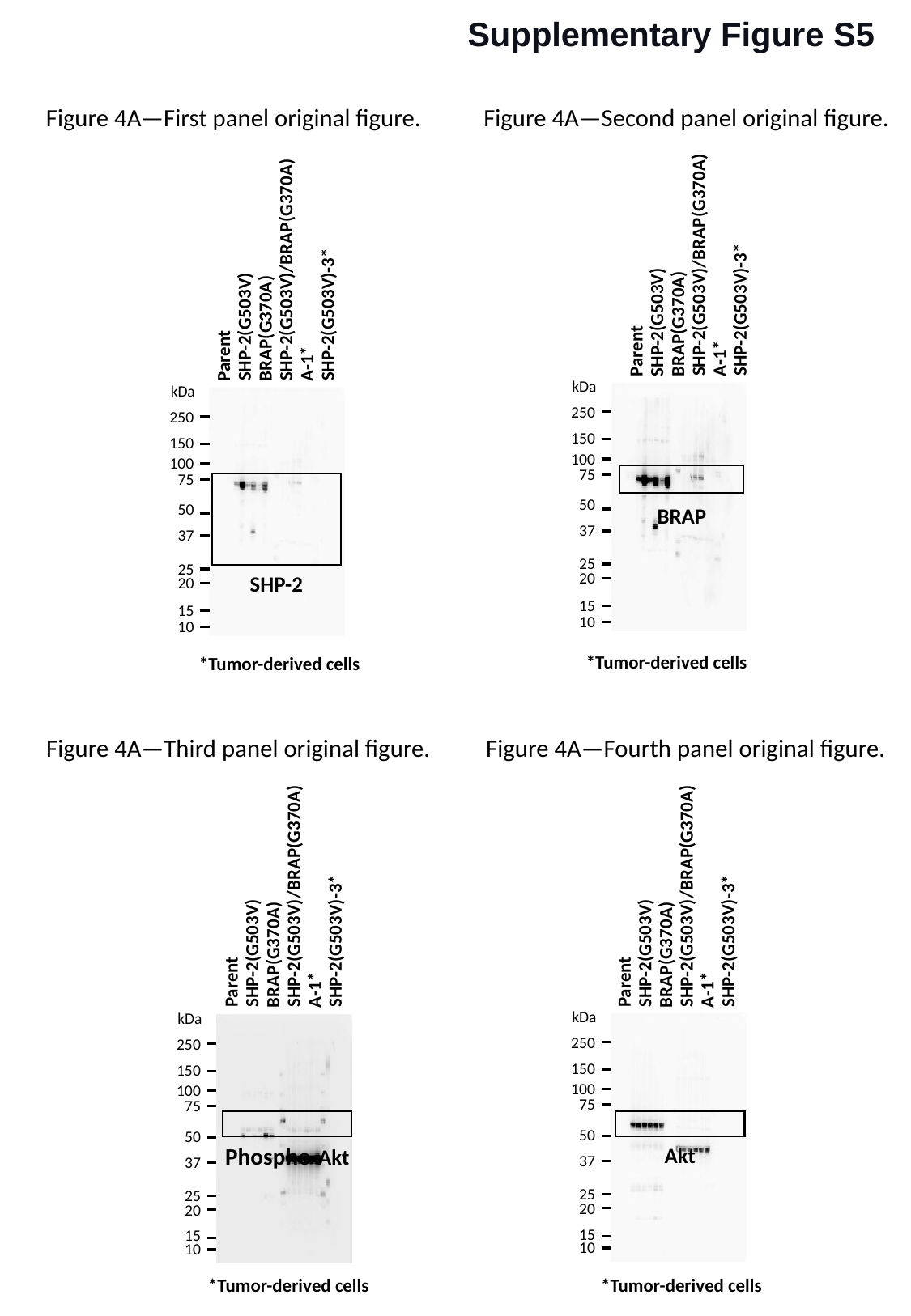

Supplementary Figure S5
Figure 4A—First panel original figure.
Figure 4A—Second panel original figure.
SHP-2(G503V)/BRAP(G370A)
SHP-2(G503V)-3*
SHP-2(G503V)
BRAP(G370A)
Parent
A-1*
kDa
250
150
100
75
50
37
25
20
15
10
BRAP
*Tumor-derived cells
SHP-2(G503V)/BRAP(G370A)
SHP-2(G503V)-3*
SHP-2(G503V)
BRAP(G370A)
Parent
A-1*
kDa
250
150
100
75
50
37
25
20
15
10
SHP-2
*Tumor-derived cells
Figure 4A—Third panel original figure.
Figure 4A—Fourth panel original figure.
SHP-2(G503V)/BRAP(G370A)
SHP-2(G503V)-3*
SHP-2(G503V)
BRAP(G370A)
Parent
A-1*
kDa
250
150
100
75
50
37
25
20
15
10
Phospho-Akt
*Tumor-derived cells
SHP-2(G503V)/BRAP(G370A)
SHP-2(G503V)-3*
SHP-2(G503V)
BRAP(G370A)
Parent
A-1*
kDa
250
150
100
75
50
37
25
20
15
10
Akt
*Tumor-derived cells

## Slide 4
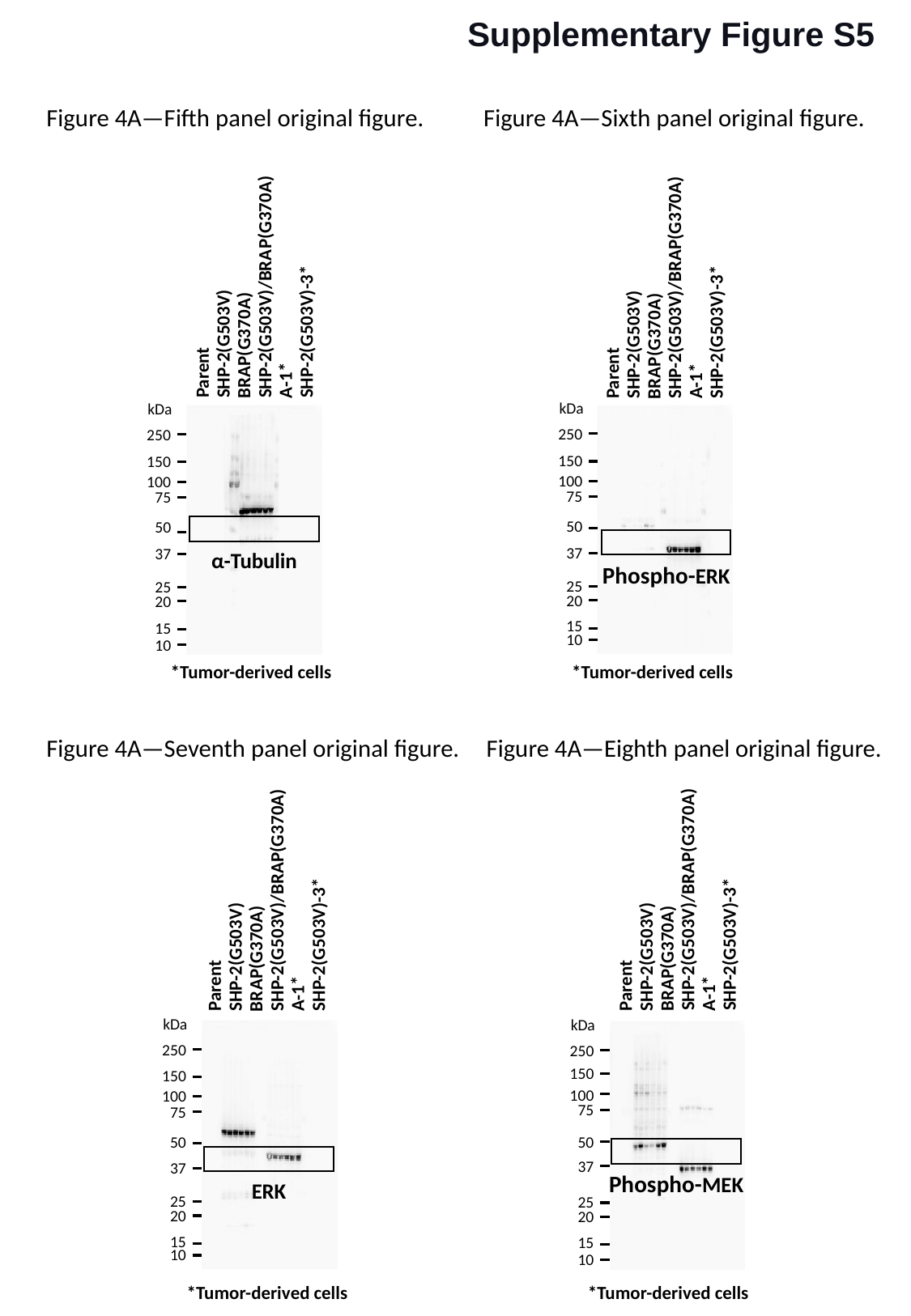

Supplementary Figure S5
Figure 4A—Fifth panel original figure.
Figure 4A—Sixth panel original figure.
SHP-2(G503V)/BRAP(G370A)
SHP-2(G503V)-3*
SHP-2(G503V)
BRAP(G370A)
Parent
A-1*
kDa
250
150
100
75
50
37
25
20
15
10
α-Tubulin
*Tumor-derived cells
SHP-2(G503V)/BRAP(G370A)
SHP-2(G503V)-3*
SHP-2(G503V)
BRAP(G370A)
Parent
A-1*
kDa
250
150
100
75
50
37
25
20
15
10
Phospho-ERK
*Tumor-derived cells
Figure 4A—Seventh panel original figure.
Figure 4A—Eighth panel original figure.
SHP-2(G503V)/BRAP(G370A)
SHP-2(G503V)-3*
SHP-2(G503V)
BRAP(G370A)
Parent
A-1*
kDa
250
150
100
75
50
37
25
20
15
10
Phospho-MEK
*Tumor-derived cells
SHP-2(G503V)/BRAP(G370A)
SHP-2(G503V)-3*
SHP-2(G503V)
BRAP(G370A)
Parent
A-1*
kDa
250
150
100
75
50
37
25
20
15
10
ERK
*Tumor-derived cells

## Slide 5
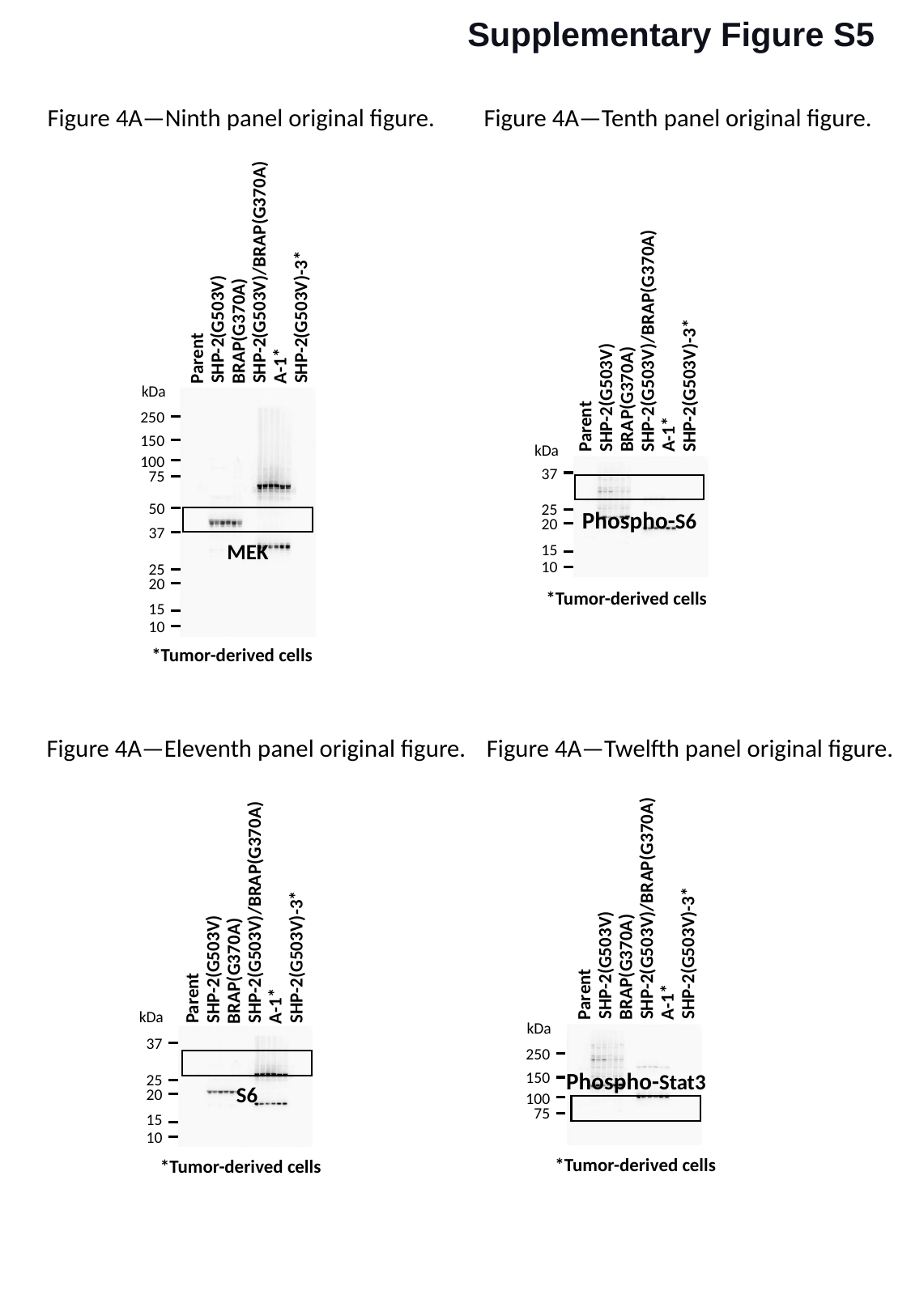

Supplementary Figure S5
Figure 4A—Ninth panel original figure.
Figure 4A—Tenth panel original figure.
SHP-2(G503V)/BRAP(G370A)
SHP-2(G503V)-3*
SHP-2(G503V)
BRAP(G370A)
Parent
A-1*
kDa
250
150
100
75
50
37
25
20
15
10
MEK
*Tumor-derived cells
SHP-2(G503V)/BRAP(G370A)
SHP-2(G503V)-3*
SHP-2(G503V)
BRAP(G370A)
Parent
A-1*
kDa
37
25
20
15
10
Phospho-S6
*Tumor-derived cells
Figure 4A—Eleventh panel original figure.
Figure 4A—Twelfth panel original figure.
SHP-2(G503V)/BRAP(G370A)
SHP-2(G503V)-3*
SHP-2(G503V)
BRAP(G370A)
Parent
A-1*
SHP-2(G503V)/BRAP(G370A)
SHP-2(G503V)-3*
SHP-2(G503V)
BRAP(G370A)
Parent
A-1*
kDa
37
25
20
15
10
S6
*Tumor-derived cells
kDa
250
150
100
75
Phospho-Stat3
*Tumor-derived cells

## Slide 6
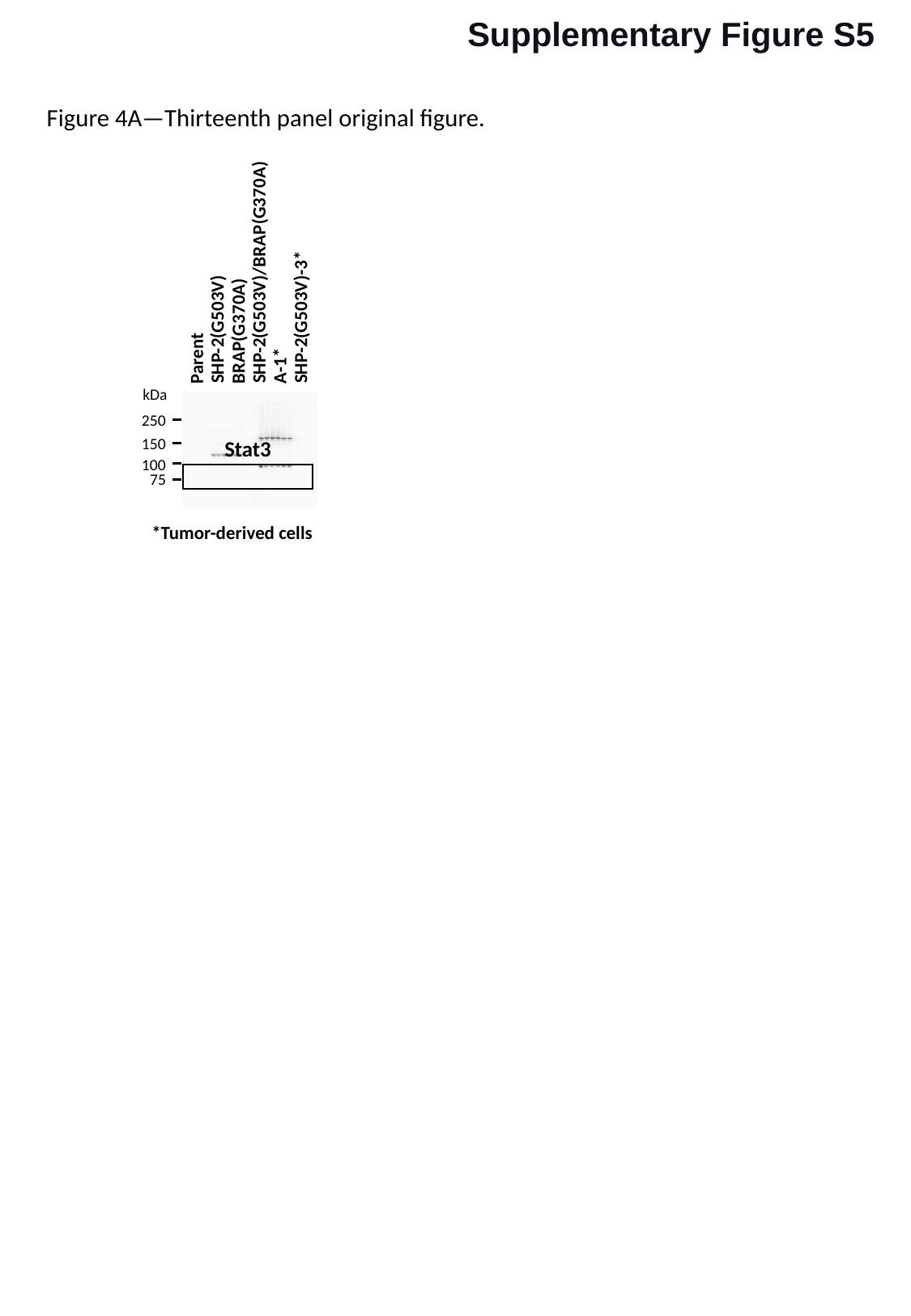

Supplementary Figure S5
Figure 4A—Thirteenth panel original figure.
SHP-2(G503V)/BRAP(G370A)
SHP-2(G503V)-3*
SHP-2(G503V)
BRAP(G370A)
Parent
A-1*
kDa
250
150
100
75
Stat3
*Tumor-derived cells

## Slide 7
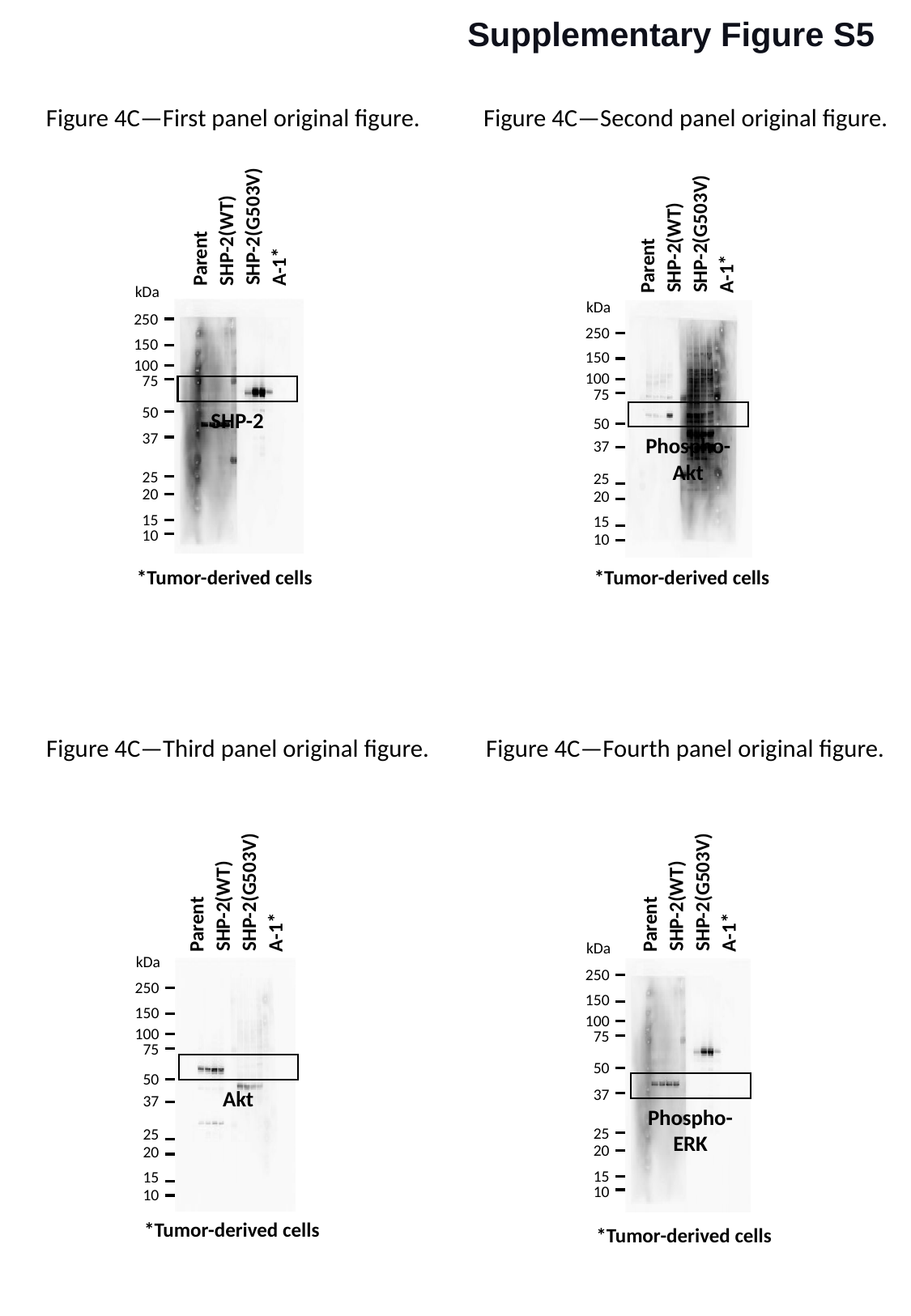

Supplementary Figure S5
Figure 4C—First panel original figure.
Figure 4C—Second panel original figure.
SHP-2(G503V)
SHP-2(WT)
Parent
A-1*
kDa
250
150
100
75
50
37
25
20
15
10
SHP-2
*Tumor-derived cells
SHP-2(G503V)
SHP-2(WT)
Parent
A-1*
kDa
250
150
100
75
50
37
25
20
15
10
Phospho-
Akt
*Tumor-derived cells
Figure 4C—Third panel original figure.
Figure 4C—Fourth panel original figure.
SHP-2(G503V)
SHP-2(WT)
Parent
A-1*
kDa
250
150
100
75
50
37
25
20
15
10
Akt
*Tumor-derived cells
SHP-2(G503V)
SHP-2(WT)
Parent
A-1*
kDa
250
150
100
75
50
37
25
20
15
10
Phospho-
ERK
*Tumor-derived cells

## Slide 8
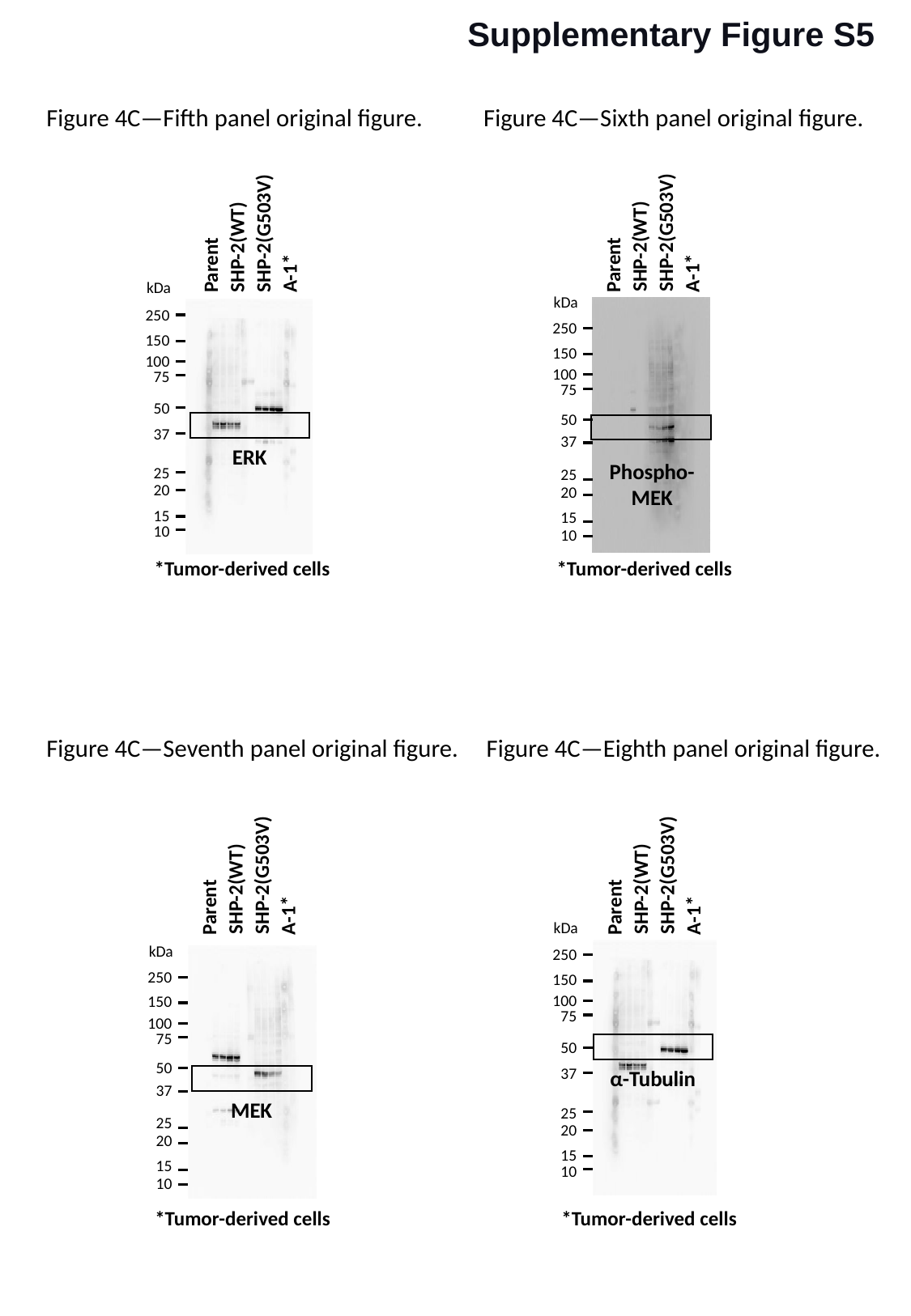

Supplementary Figure S5
Figure 4C—Fifth panel original figure.
Figure 4C—Sixth panel original figure.
SHP-2(G503V)
SHP-2(WT)
Parent
A-1*
kDa
250
150
100
75
50
37
25
20
15
10
Phospho-
MEK
*Tumor-derived cells
SHP-2(G503V)
SHP-2(WT)
Parent
A-1*
kDa
250
150
100
75
50
37
25
20
15
10
ERK
*Tumor-derived cells
Figure 4C—Seventh panel original figure.
Figure 4C—Eighth panel original figure.
SHP-2(G503V)
SHP-2(WT)
Parent
A-1*
kDa
250
150
100
75
50
37
25
20
15
10
MEK
*Tumor-derived cells
SHP-2(G503V)
SHP-2(WT)
Parent
A-1*
kDa
250
150
100
75
50
37
25
20
15
10
α-Tubulin
*Tumor-derived cells

## Slide 9
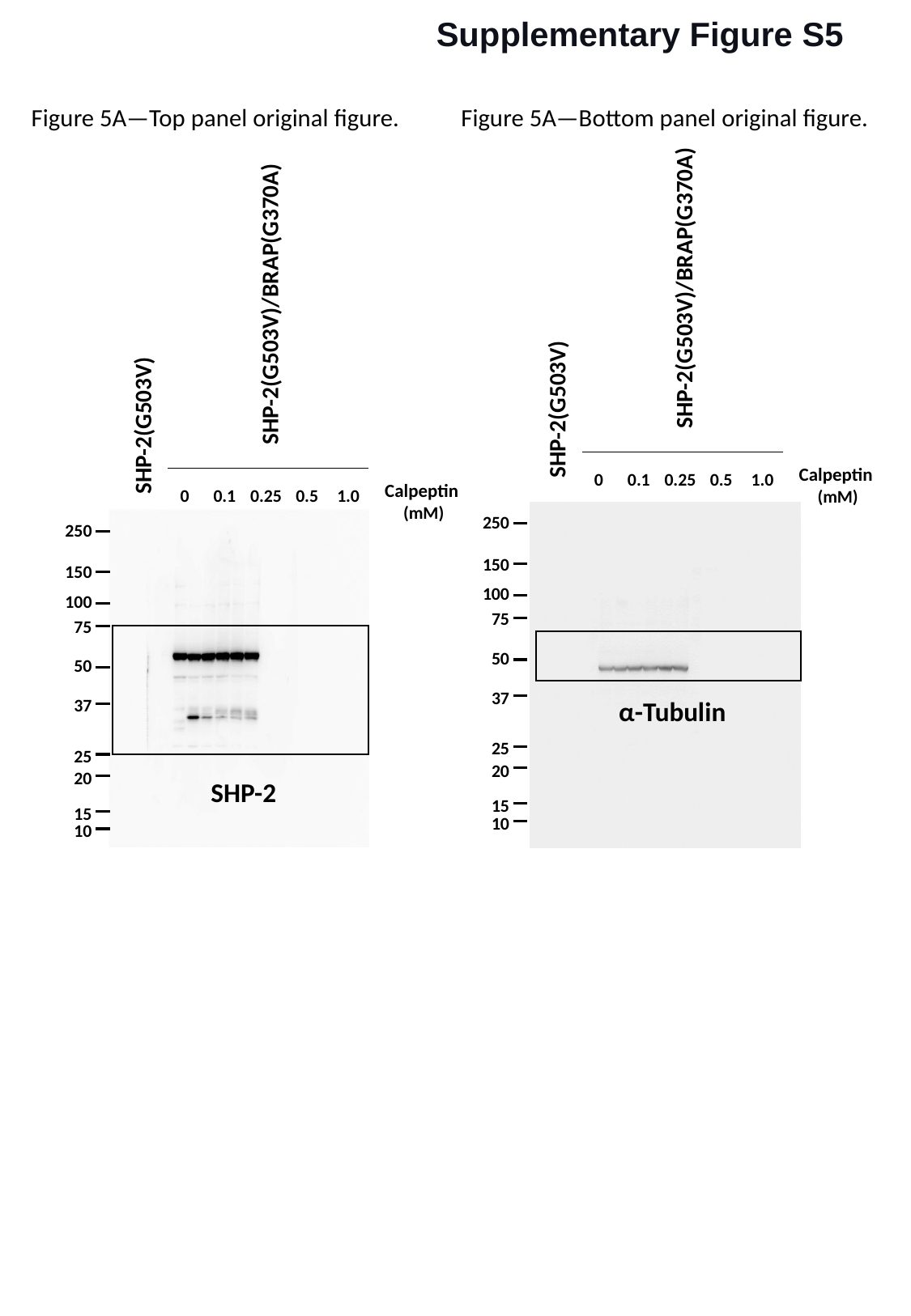

Supplementary Figure S5
Figure 5A—Top panel original figure.
Figure 5A—Bottom panel original figure.
SHP-2(G503V)/BRAP(G370A)
SHP-2(G503V)
Calpeptin
(mM)
0
0.1
0.25
0.5
1.0
250
150
100
75
50
37
25
20
15
10
α-Tubulin
SHP-2(G503V)/BRAP(G370A)
SHP-2(G503V)
Calpeptin
(mM)
0
0.1
0.25
0.5
1.0
250
150
100
75
50
37
25
20
15
10
SHP-2

## Slide 10
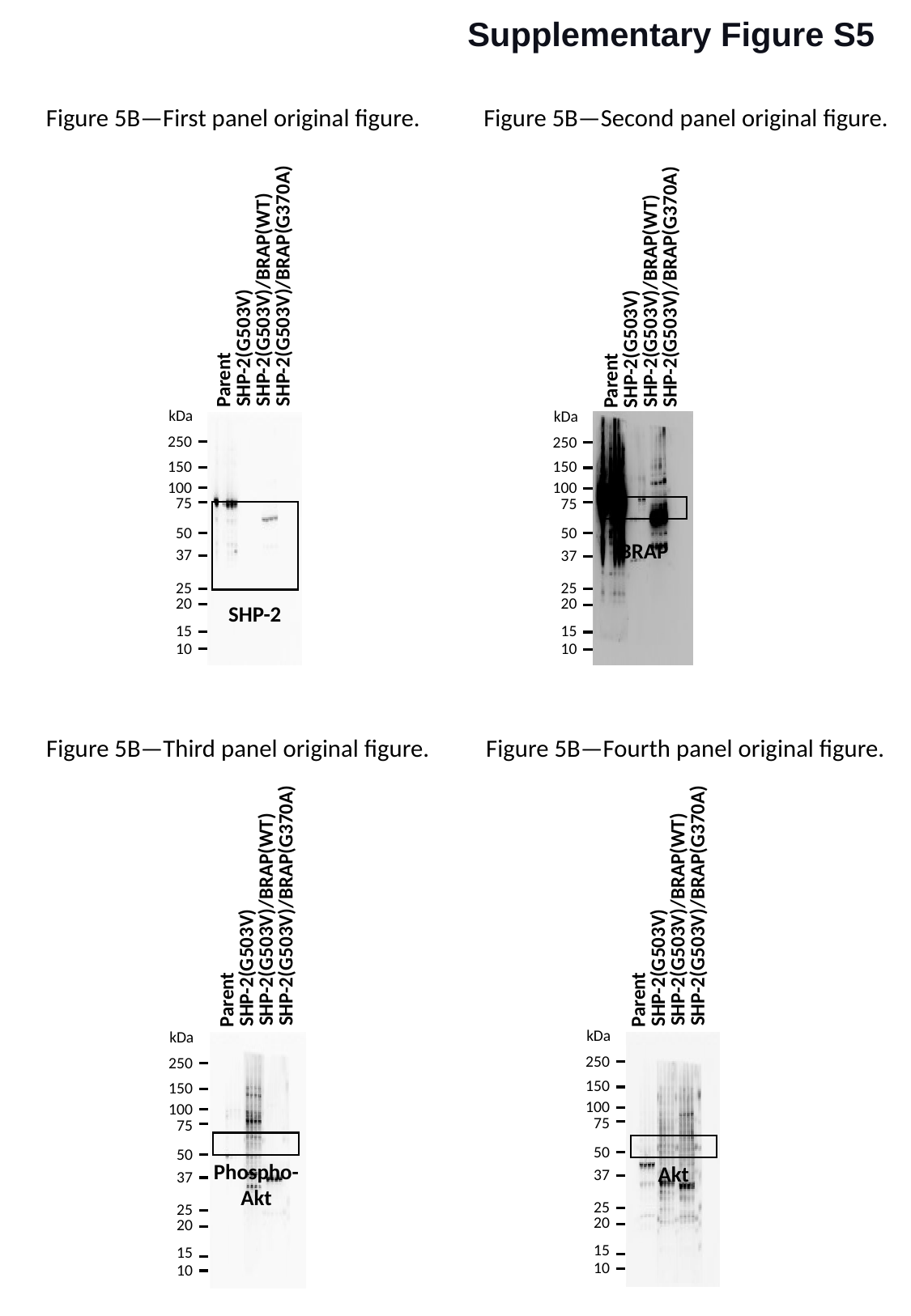

Supplementary Figure S5
Figure 5B—First panel original figure.
Figure 5B—Second panel original figure.
SHP-2(G503V)/BRAP(G370A)
SHP-2(G503V)/BRAP(WT)
SHP-2(G503V)
Parent
kDa
250
150
100
75
50
37
25
20
15
10
SHP-2
SHP-2(G503V)/BRAP(G370A)
SHP-2(G503V)/BRAP(WT)
SHP-2(G503V)
Parent
kDa
250
150
100
75
50
37
25
20
15
10
BRAP
Figure 5B—Third panel original figure.
Figure 5B—Fourth panel original figure.
SHP-2(G503V)/BRAP(G370A)
SHP-2(G503V)/BRAP(WT)
SHP-2(G503V)
Parent
kDa
250
150
100
75
50
37
25
20
15
10
Phospho-
Akt
SHP-2(G503V)/BRAP(G370A)
SHP-2(G503V)/BRAP(WT)
SHP-2(G503V)
Parent
kDa
250
150
100
75
50
37
25
20
15
10
Akt

## Slide 11
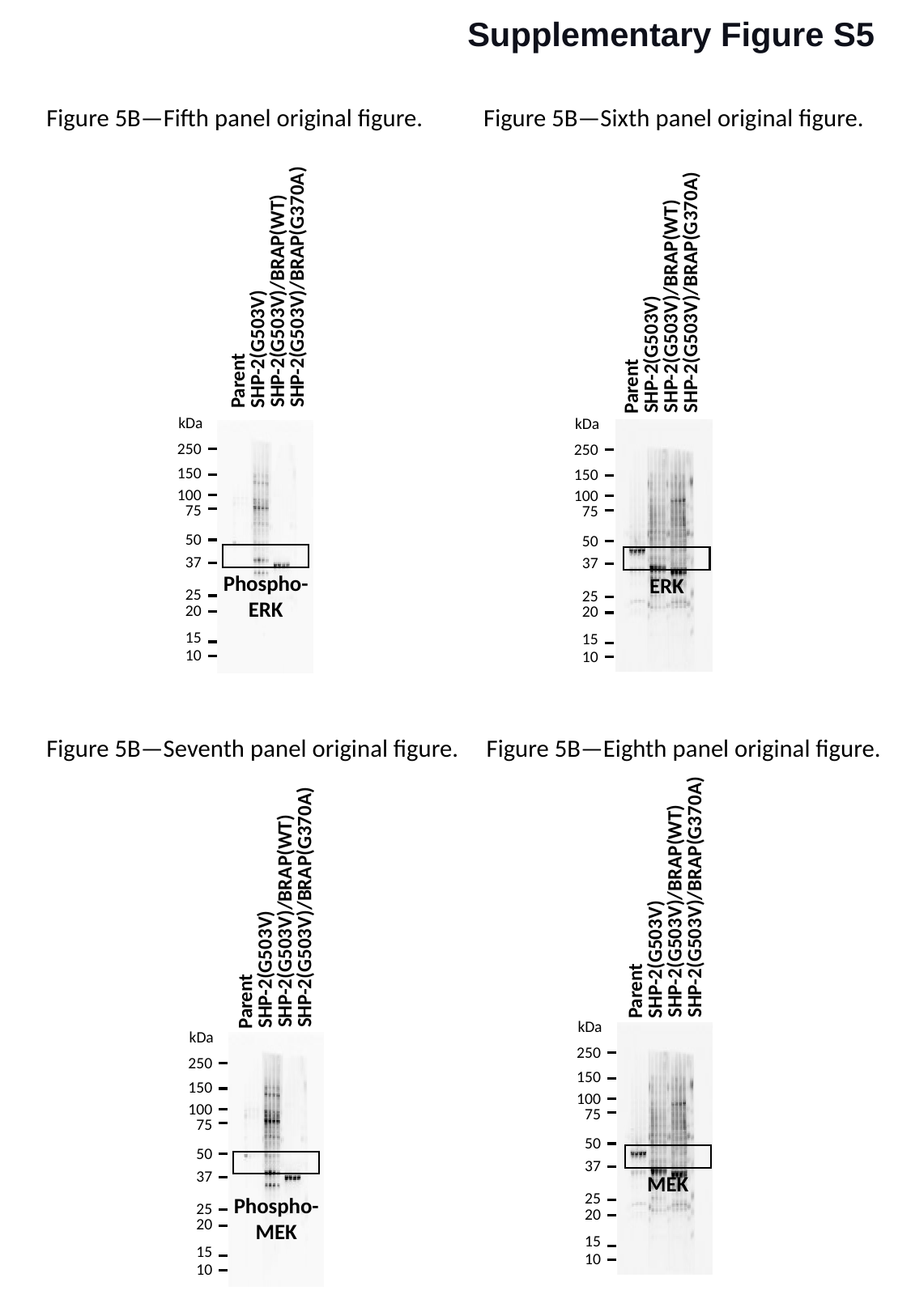

Supplementary Figure S5
Figure 5B—Fifth panel original figure.
Figure 5B—Sixth panel original figure.
SHP-2(G503V)/BRAP(G370A)
SHP-2(G503V)/BRAP(WT)
SHP-2(G503V)
Parent
kDa
250
150
100
75
50
37
25
20
15
10
Phospho-
ERK
SHP-2(G503V)/BRAP(G370A)
SHP-2(G503V)/BRAP(WT)
SHP-2(G503V)
Parent
kDa
250
150
100
75
50
37
25
20
15
10
ERK
Figure 5B—Seventh panel original figure.
Figure 5B—Eighth panel original figure.
SHP-2(G503V)/BRAP(G370A)
SHP-2(G503V)/BRAP(WT)
SHP-2(G503V)
Parent
kDa
250
150
100
75
50
37
25
20
15
10
MEK
SHP-2(G503V)/BRAP(G370A)
SHP-2(G503V)/BRAP(WT)
SHP-2(G503V)
Parent
kDa
250
150
100
75
50
37
25
20
15
10
Phospho-
MEK

## Slide 12
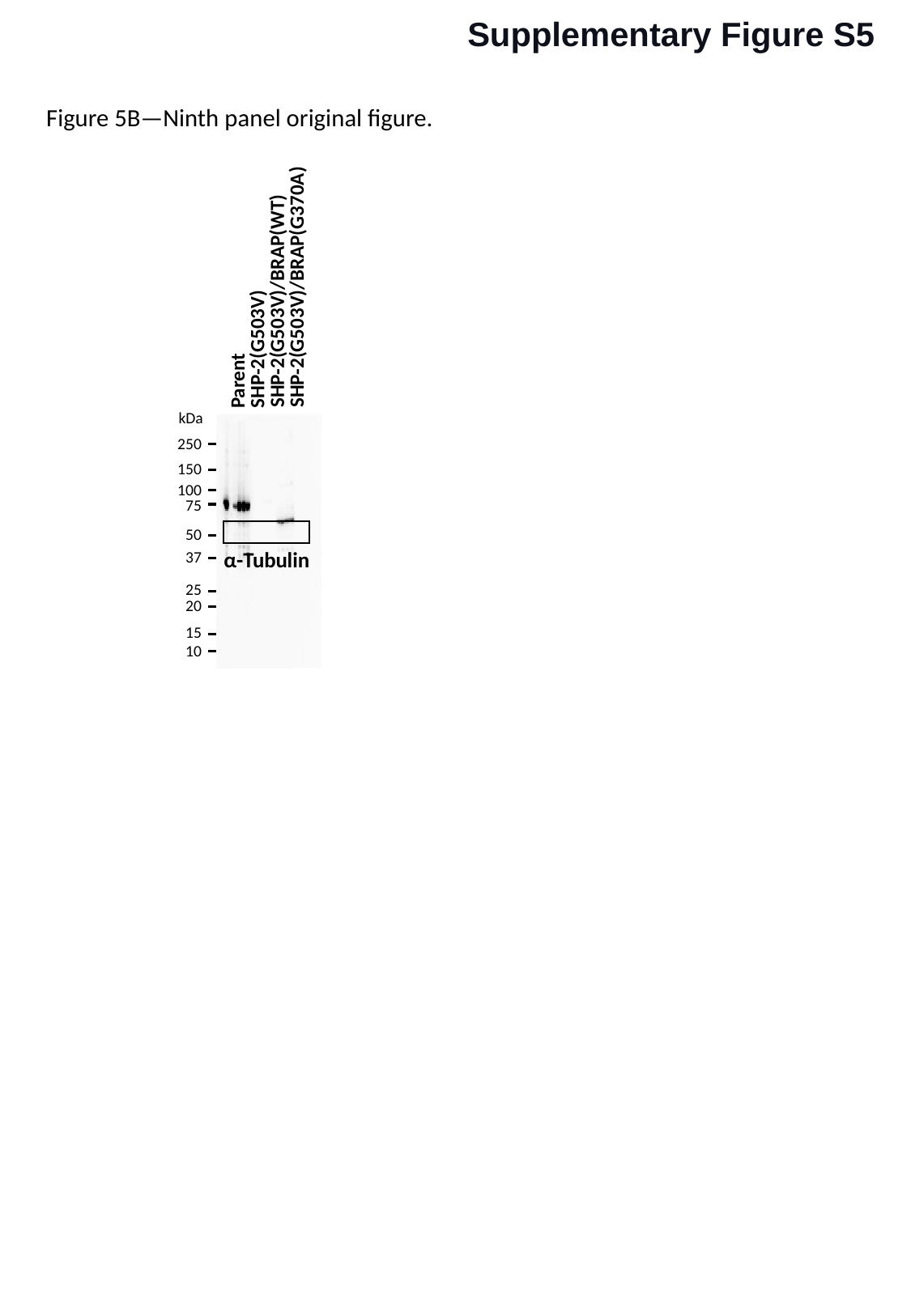

Supplementary Figure S5
Figure 5B—Ninth panel original figure.
SHP-2(G503V)/BRAP(G370A)
SHP-2(G503V)/BRAP(WT)
SHP-2(G503V)
Parent
kDa
250
150
100
75
50
37
25
20
15
10
α-Tubulin

## Slide 13
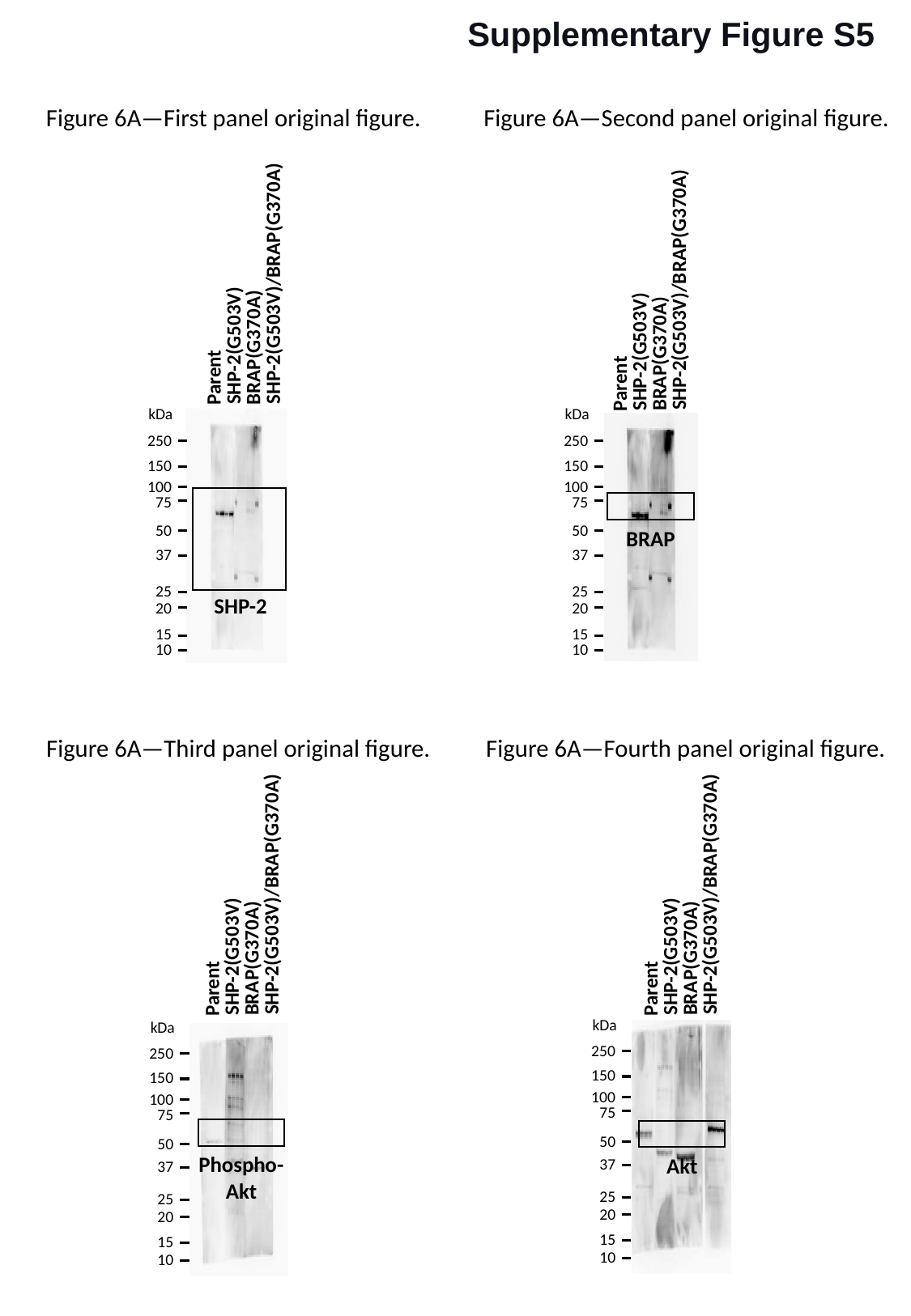

Supplementary Figure S5
Figure 6A—First panel original figure.
Figure 6A—Second panel original figure.
SHP-2(G503V)/BRAP(G370A)
SHP-2(G503V)
BRAP(G370A)
Parent
kDa
250
150
100
75
50
37
25
20
15
10
SHP-2
SHP-2(G503V)/BRAP(G370A)
SHP-2(G503V)
BRAP(G370A)
Parent
kDa
250
150
100
75
50
37
25
20
15
10
BRAP
Figure 6A—Third panel original figure.
Figure 6A—Fourth panel original figure.
SHP-2(G503V)/BRAP(G370A)
SHP-2(G503V)
BRAP(G370A)
Parent
kDa
250
150
100
75
50
37
25
20
15
10
Phospho-
Akt
SHP-2(G503V)/BRAP(G370A)
SHP-2(G503V)
BRAP(G370A)
Parent
kDa
250
150
100
75
50
37
25
20
15
10
Akt

## Slide 14
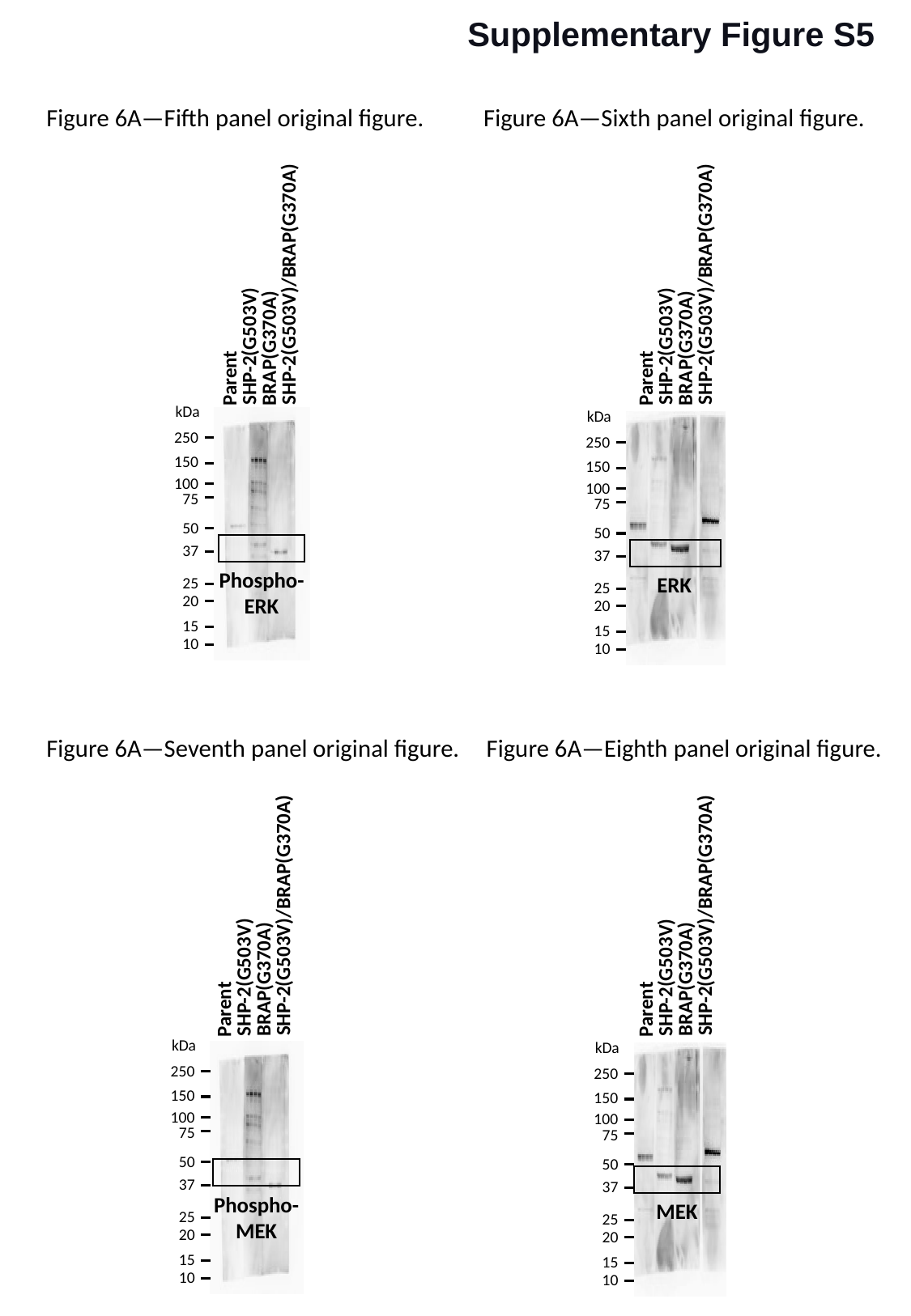

Supplementary Figure S5
Figure 6A—Fifth panel original figure.
Figure 6A—Sixth panel original figure.
SHP-2(G503V)/BRAP(G370A)
SHP-2(G503V)
BRAP(G370A)
Parent
kDa
250
150
100
75
50
37
25
20
15
10
Phospho-
ERK
SHP-2(G503V)/BRAP(G370A)
SHP-2(G503V)
BRAP(G370A)
Parent
kDa
250
150
100
75
50
37
25
20
15
10
ERK
Figure 6A—Seventh panel original figure.
Figure 6A—Eighth panel original figure.
SHP-2(G503V)/BRAP(G370A)
SHP-2(G503V)
BRAP(G370A)
Parent
kDa
250
150
100
75
50
37
25
20
15
10
Phospho-
MEK
SHP-2(G503V)/BRAP(G370A)
SHP-2(G503V)
BRAP(G370A)
Parent
kDa
250
150
100
75
50
37
25
20
15
10
MEK

## Slide 15
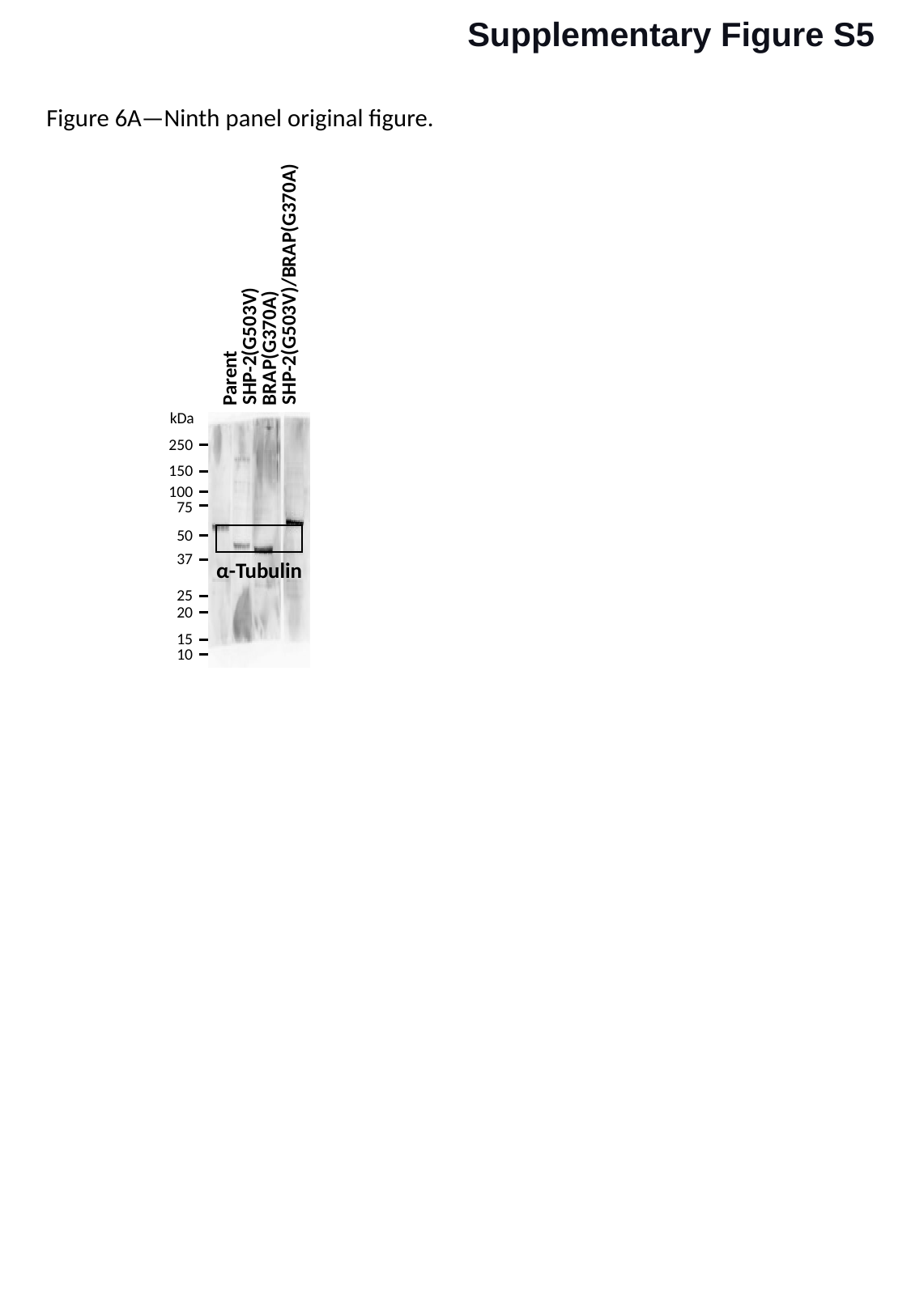

Supplementary Figure S5
Figure 6A—Ninth panel original figure.
SHP-2(G503V)/BRAP(G370A)
SHP-2(G503V)
BRAP(G370A)
Parent
kDa
250
150
100
75
50
37
25
20
15
10
α-Tubulin

## Slide 16
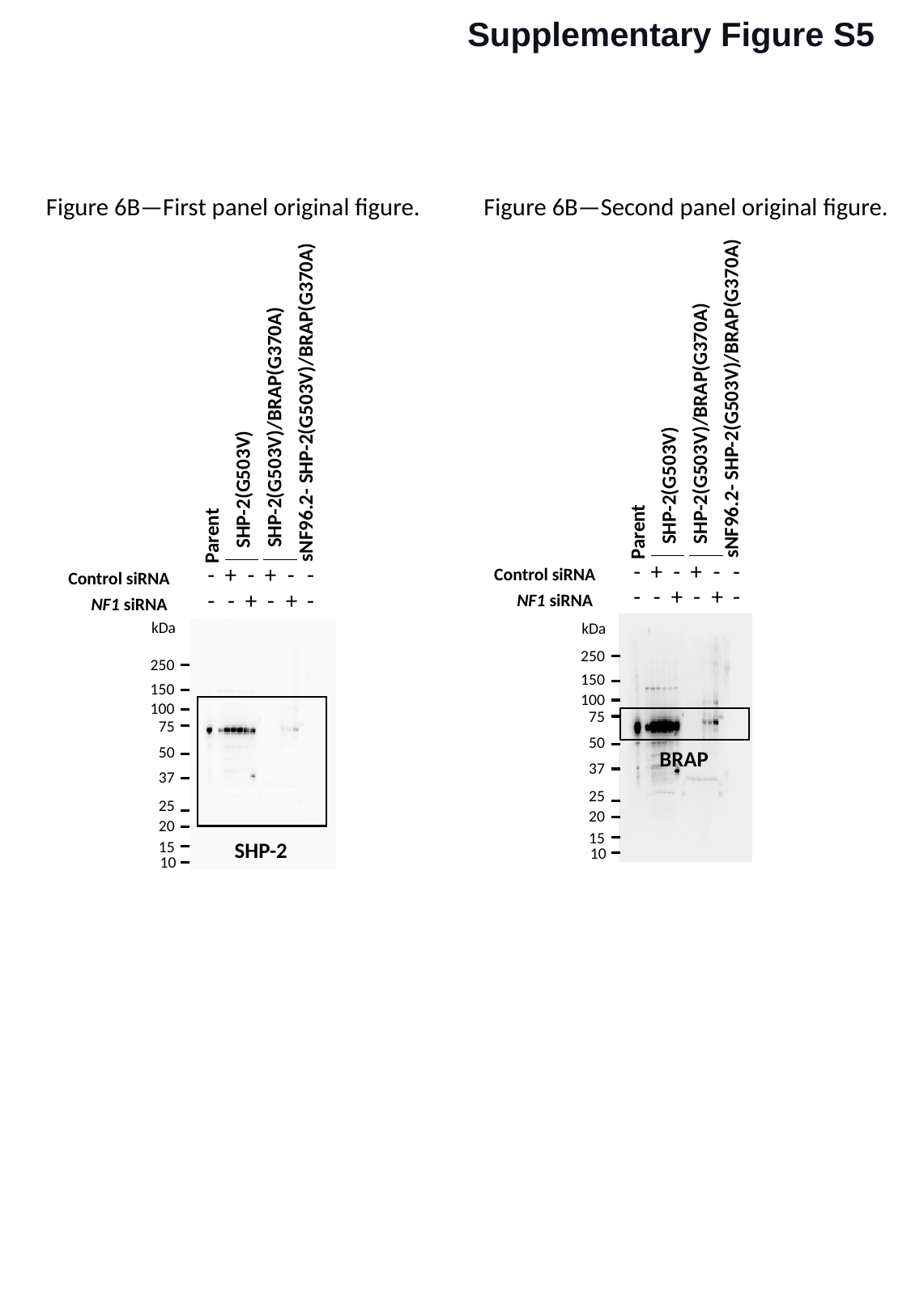

Supplementary Figure S5
Figure 6B—First panel original figure.
Figure 6B—Second panel original figure.
sNF96.2- SHP-2(G503V)/BRAP(G370A)
SHP-2(G503V)/BRAP(G370A)
SHP-2(G503V)
Parent
-
+
-
+
-
-
Control siRNA
-
-
+
-
+
-
NF1 siRNA
kDa
250
150
100
75
50
37
25
20
15
10
BRAP
sNF96.2- SHP-2(G503V)/BRAP(G370A)
SHP-2(G503V)/BRAP(G370A)
SHP-2(G503V)
Parent
-
+
-
+
-
-
Control siRNA
-
-
+
-
+
-
NF1 siRNA
kDa
250
150
100
75
50
37
25
20
15
10
SHP-2

## Slide 17
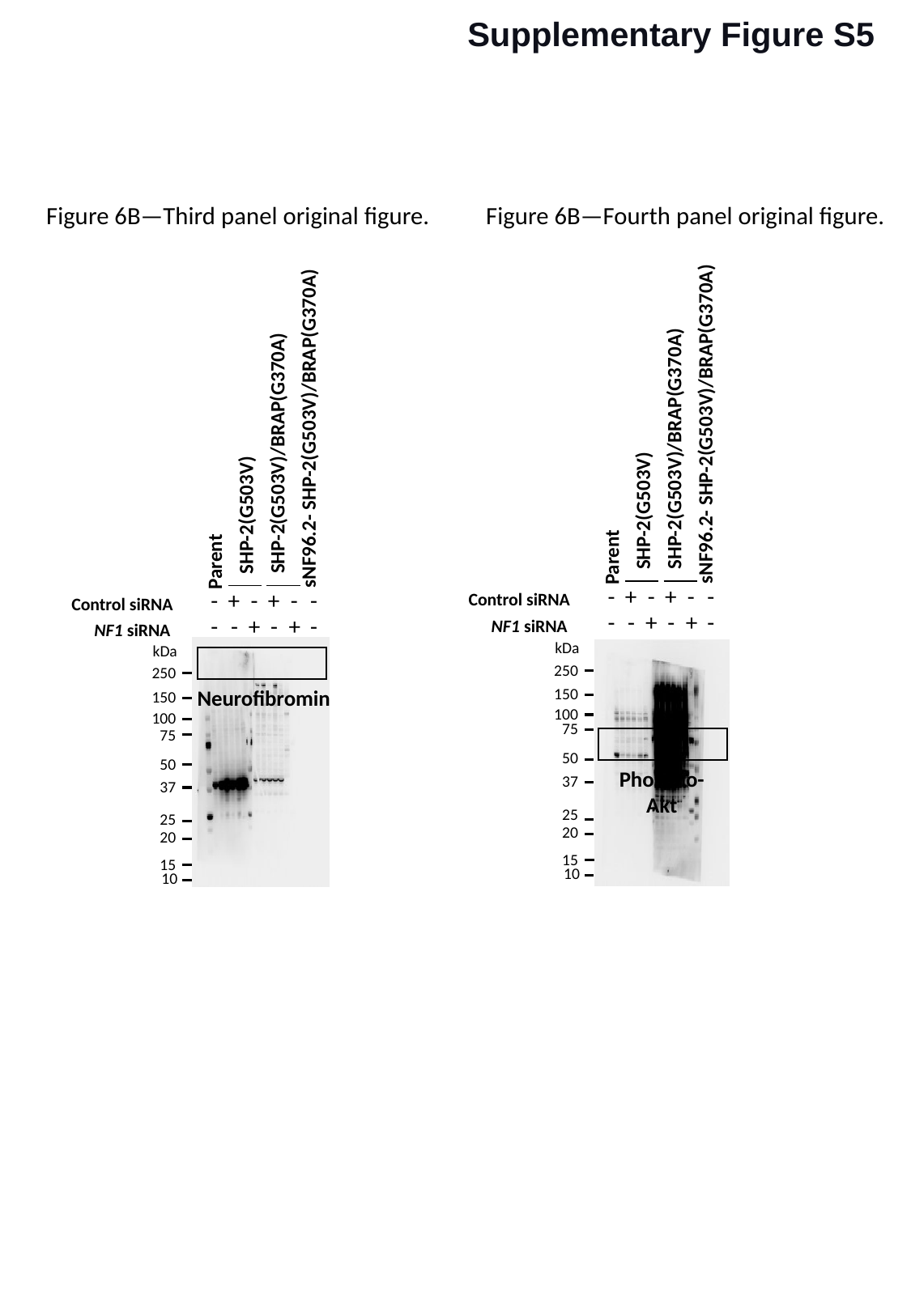

Supplementary Figure S5
Figure 6B—Third panel original figure.
Figure 6B—Fourth panel original figure.
sNF96.2- SHP-2(G503V)/BRAP(G370A)
SHP-2(G503V)/BRAP(G370A)
SHP-2(G503V)
Parent
-
+
-
+
-
-
Control siRNA
-
-
+
-
+
-
NF1 siRNA
sNF96.2- SHP-2(G503V)/BRAP(G370A)
SHP-2(G503V)/BRAP(G370A)
SHP-2(G503V)
Parent
-
+
-
+
-
-
Control siRNA
-
-
+
-
+
-
NF1 siRNA
kDa
250
150
100
75
50
37
25
20
15
10
Neurofibromin
kDa
250
150
100
75
50
37
25
20
15
10
Phospho-
Akt

## Slide 18
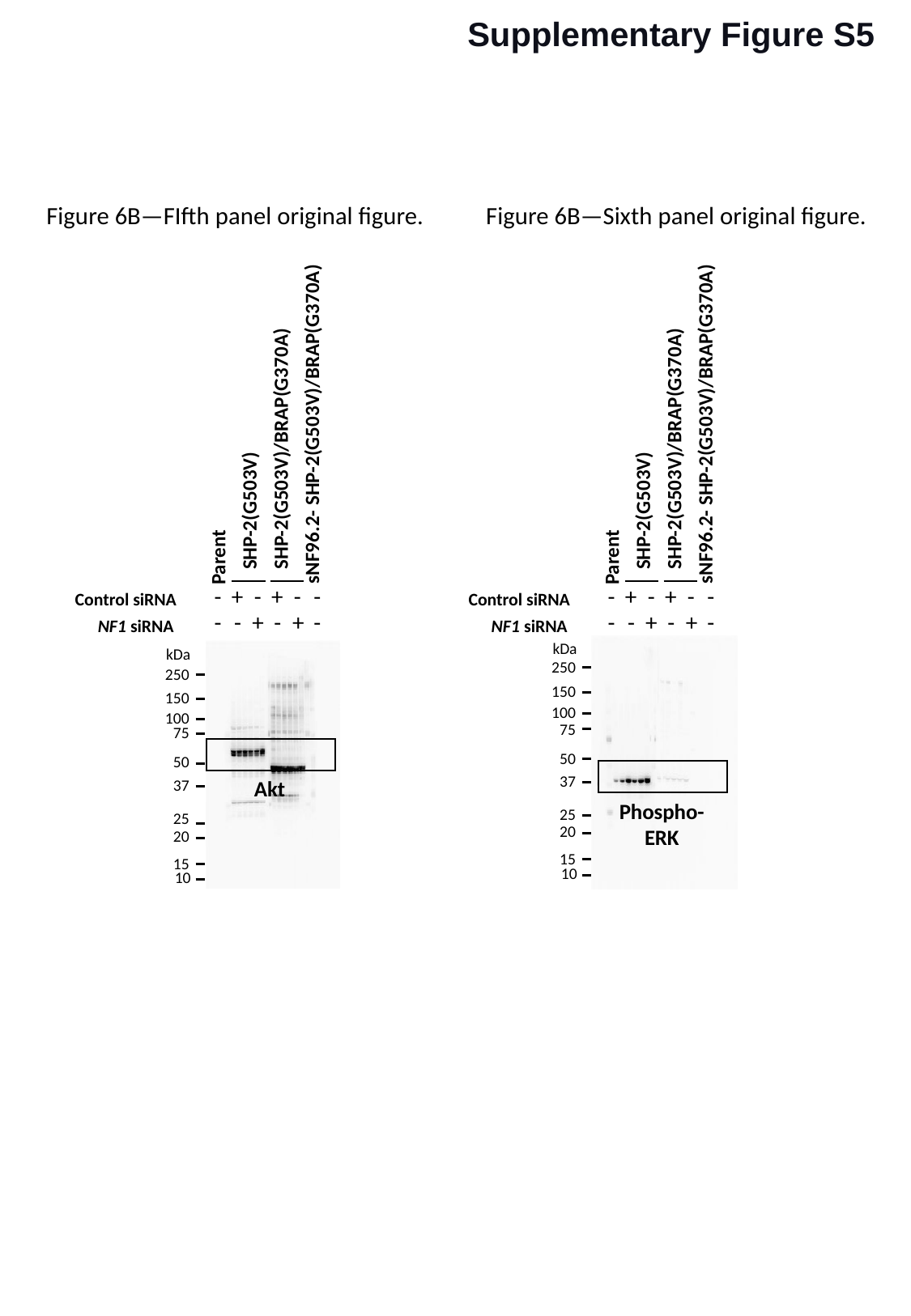

Supplementary Figure S5
Figure 6B—FIfth panel original figure.
Figure 6B—Sixth panel original figure.
sNF96.2- SHP-2(G503V)/BRAP(G370A)
SHP-2(G503V)/BRAP(G370A)
SHP-2(G503V)
Parent
-
+
-
+
-
-
Control siRNA
-
-
+
-
+
-
NF1 siRNA
kDa
250
150
100
75
50
37
25
20
15
10
Akt
sNF96.2- SHP-2(G503V)/BRAP(G370A)
SHP-2(G503V)/BRAP(G370A)
SHP-2(G503V)
Parent
-
+
-
+
-
-
Control siRNA
-
-
+
-
+
-
NF1 siRNA
kDa
250
150
100
75
50
37
25
20
15
10
Phospho-
ERK

## Slide 19
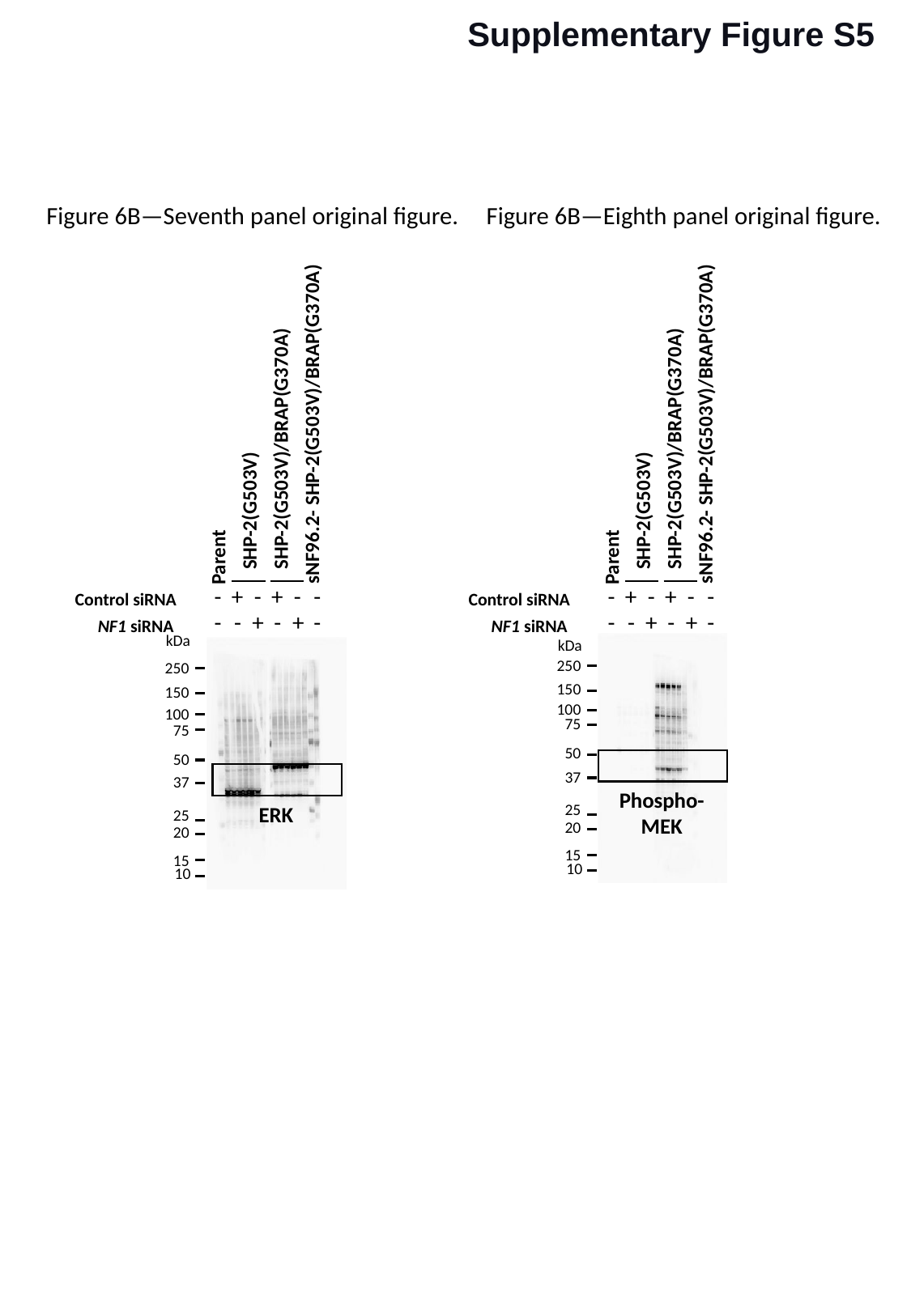

Supplementary Figure S5
Figure 6B—Seventh panel original figure.
Figure 6B—Eighth panel original figure.
sNF96.2- SHP-2(G503V)/BRAP(G370A)
SHP-2(G503V)/BRAP(G370A)
SHP-2(G503V)
Parent
-
+
-
+
-
-
Control siRNA
-
-
+
-
+
-
NF1 siRNA
kDa
250
150
100
75
50
37
25
20
15
10
ERK
sNF96.2- SHP-2(G503V)/BRAP(G370A)
SHP-2(G503V)/BRAP(G370A)
SHP-2(G503V)
Parent
-
+
-
+
-
-
Control siRNA
-
-
+
-
+
-
NF1 siRNA
kDa
250
150
100
75
50
37
25
20
15
10
Phospho-
MEK

## Slide 20
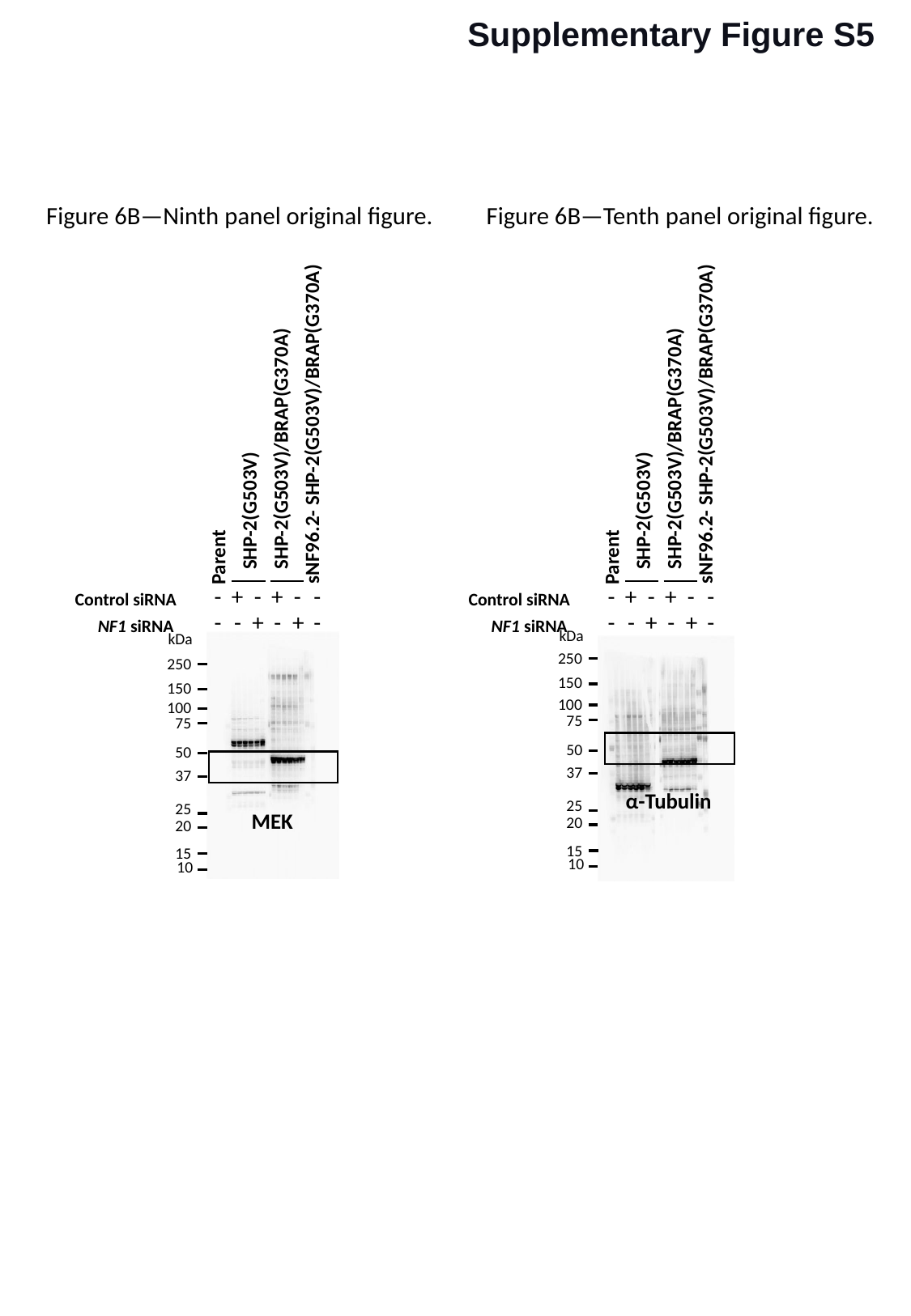

Supplementary Figure S5
Figure 6B—Ninth panel original figure.
Figure 6B—Tenth panel original figure.
sNF96.2- SHP-2(G503V)/BRAP(G370A)
SHP-2(G503V)/BRAP(G370A)
SHP-2(G503V)
Parent
-
+
-
+
-
-
Control siRNA
-
-
+
-
+
-
NF1 siRNA
sNF96.2- SHP-2(G503V)/BRAP(G370A)
SHP-2(G503V)/BRAP(G370A)
SHP-2(G503V)
Parent
-
+
-
+
-
-
Control siRNA
-
-
+
-
+
-
NF1 siRNA
kDa
250
150
100
75
50
37
25
20
15
10
α-Tubulin
kDa
250
150
100
75
50
37
25
20
15
10
MEK
