# Supplementary material for: Mutation of PTPN11 (Encoding SHP-2) Promotes MEK Activation and Malignant Progression in Neurofibromin-Deficient Cells in a Manner Sensitive to BRAP Mutation
Source: Cancers (Basel). 2022 May 12;14(10):2377. doi: 10.3390/cancers14102377 (PMC9140047; doi:10.3390/cancers14102377)
Supplement: Supplementary file 1 [file cancers-14-02377-s001.zip › Figures S1-S4.pptx]

## Slide 1
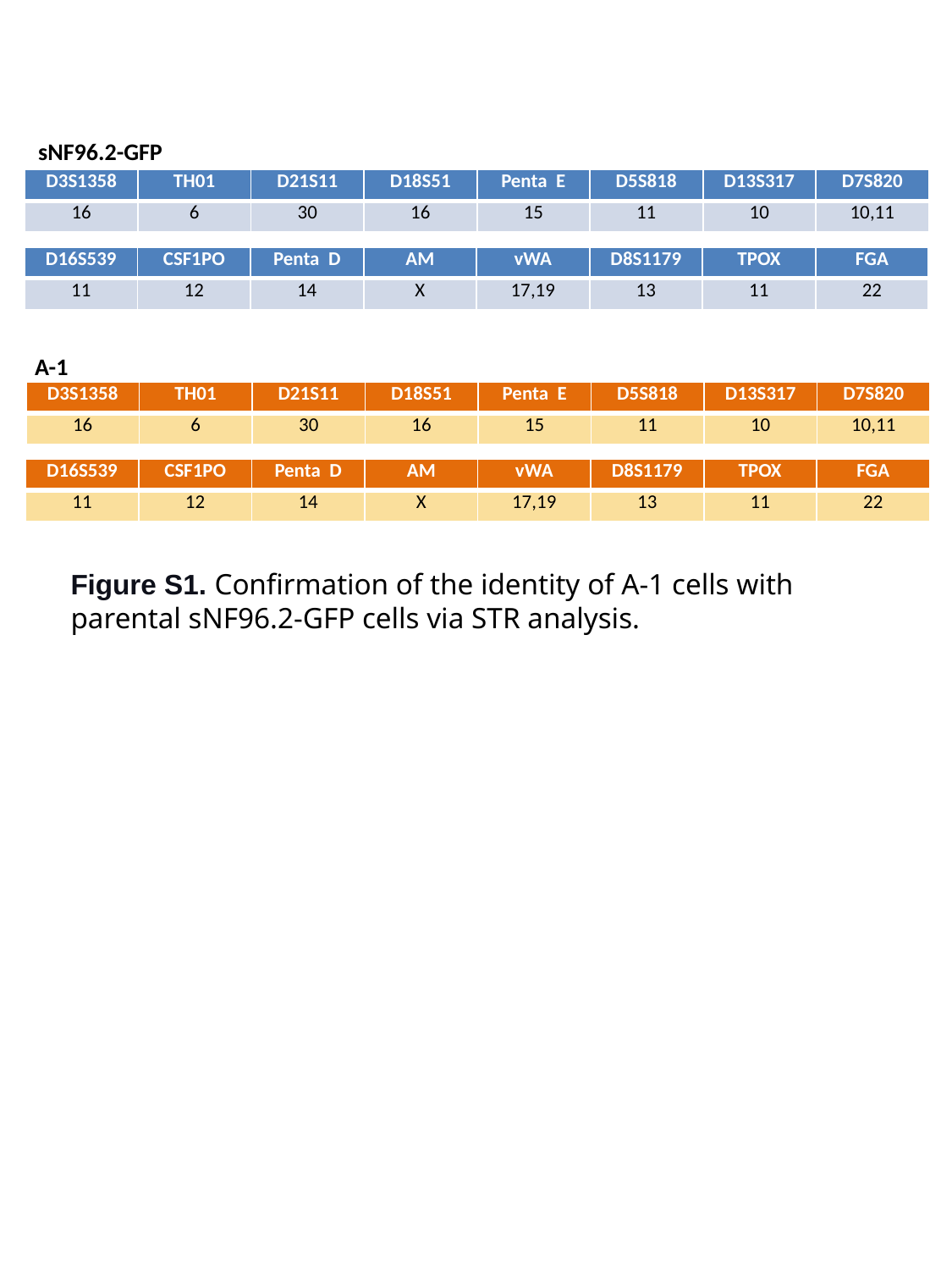

sNF96.2-GFP
| D3S1358 | TH01 | D21S11 | D18S51 | Penta E | D5S818 | D13S317 | D7S820 |
| --- | --- | --- | --- | --- | --- | --- | --- |
| 16 | 6 | 30 | 16 | 15 | 11 | 10 | 10,11 |
| D16S539 | CSF1PO | Penta D | AM | vWA | D8S1179 | TPOX | FGA |
| --- | --- | --- | --- | --- | --- | --- | --- |
| 11 | 12 | 14 | X | 17,19 | 13 | 11 | 22 |
A-1
| D3S1358 | TH01 | D21S11 | D18S51 | Penta E | D5S818 | D13S317 | D7S820 |
| --- | --- | --- | --- | --- | --- | --- | --- |
| 16 | 6 | 30 | 16 | 15 | 11 | 10 | 10,11 |
| D16S539 | CSF1PO | Penta D | AM | vWA | D8S1179 | TPOX | FGA |
| --- | --- | --- | --- | --- | --- | --- | --- |
| 11 | 12 | 14 | X | 17,19 | 13 | 11 | 22 |
Figure S1. Confirmation of the identity of A-1 cells with parental sNF96.2-GFP cells via STR analysis.

## Slide 2
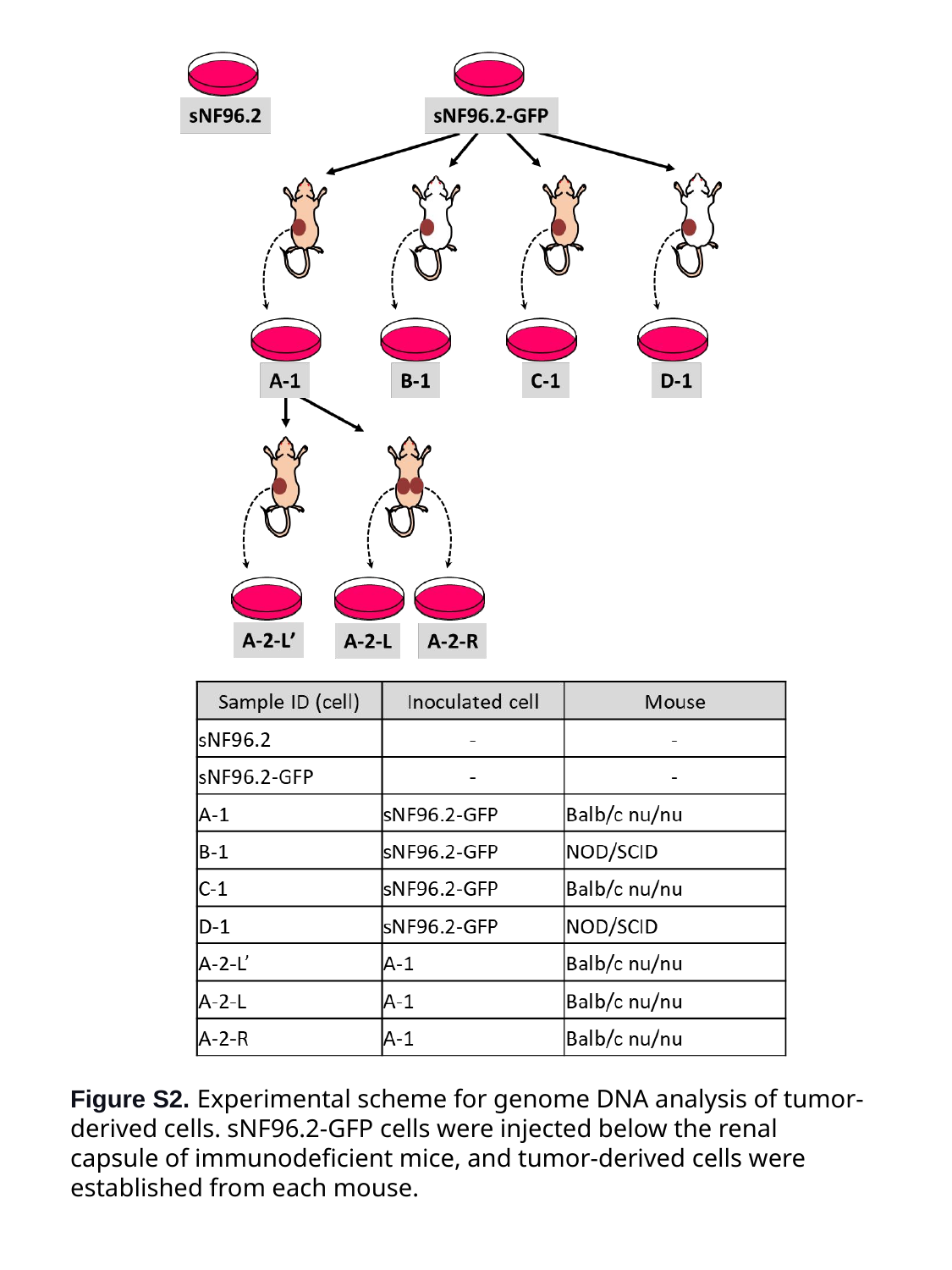

Figure S2. Experimental scheme for genome DNA analysis of tumor-derived cells. sNF96.2-GFP cells were injected below the renal capsule of immunodeficient mice, and tumor-derived cells were established from each mouse.

## Slide 3
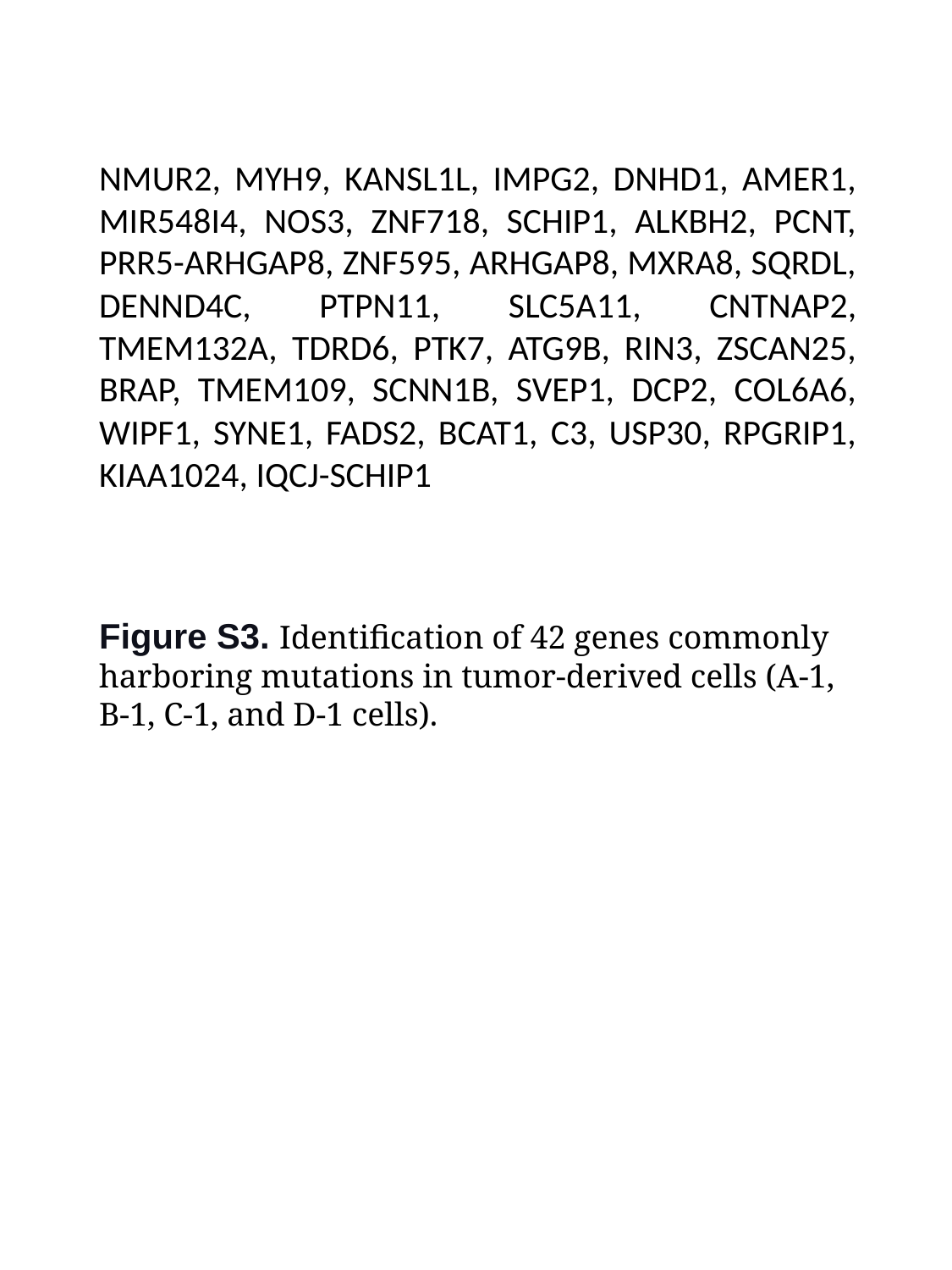

NMUR2, MYH9, KANSL1L, IMPG2, DNHD1, AMER1, MIR548I4, NOS3, ZNF718, SCHIP1, ALKBH2, PCNT, PRR5-ARHGAP8, ZNF595, ARHGAP8, MXRA8, SQRDL, DENND4C, PTPN11, SLC5A11, CNTNAP2, TMEM132A, TDRD6, PTK7, ATG9B, RIN3, ZSCAN25, BRAP, TMEM109, SCNN1B, SVEP1, DCP2, COL6A6, WIPF1, SYNE1, FADS2, BCAT1, C3, USP30, RPGRIP1, KIAA1024, IQCJ-SCHIP1
Figure S3. Identification of 42 genes commonly harboring mutations in tumor-derived cells (A-1, B-1, C-1, and D-1 cells).

## Slide 4
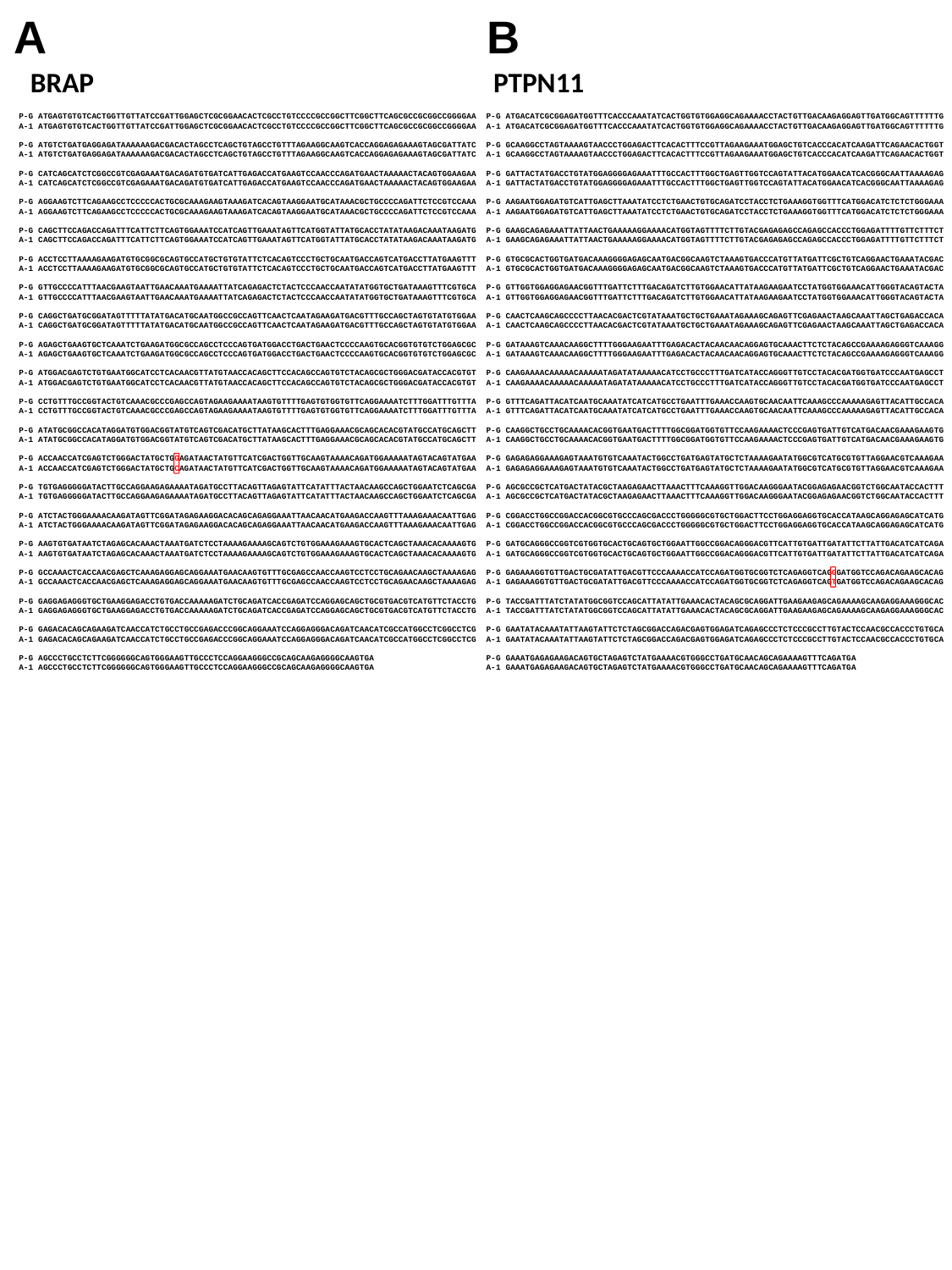

A
B
BRAP
P-G ATGAGTGTGTCACTGGTTGTTATCCGATTGGAGCTCGCGGAACACTCGCCTGTCCCCGCCGGCTTCGGCTTCAGCGCCGCGGCCGGGGAA
A-1 ATGAGTGTGTCACTGGTTGTTATCCGATTGGAGCTCGCGGAACACTCGCCTGTCCCCGCCGGCTTCGGCTTCAGCGCCGCGGCCGGGGAA
P-G ATGTCTGATGAGGAGATAAAAAAGACGACACTAGCCTCAGCTGTAGCCTGTTTAGAAGGCAAGTCACCAGGAGAGAAAGTAGCGATTATC
A-1 ATGTCTGATGAGGAGATAAAAAAGACGACACTAGCCTCAGCTGTAGCCTGTTTAGAAGGCAAGTCACCAGGAGAGAAAGTAGCGATTATC
P-G CATCAGCATCTCGGCCGTCGAGAAATGACAGATGTGATCATTGAGACCATGAAGTCCAACCCAGATGAACTAAAAACTACAGTGGAAGAA
A-1 CATCAGCATCTCGGCCGTCGAGAAATGACAGATGTGATCATTGAGACCATGAAGTCCAACCCAGATGAACTAAAAACTACAGTGGAAGAA
P-G AGGAAGTCTTCAGAAGCCTCCCCCACTGCGCAAAGAAGTAAAGATCACAGTAAGGAATGCATAAACGCTGCCCCAGATTCTCCGTCCAAA
A-1 AGGAAGTCTTCAGAAGCCTCCCCCACTGCGCAAAGAAGTAAAGATCACAGTAAGGAATGCATAAACGCTGCCCCAGATTCTCCGTCCAAA
P-G CAGCTTCCAGACCAGATTTCATTCTTCAGTGGAAATCCATCAGTTGAAATAGTTCATGGTATTATGCACCTATATAAGACAAATAAGATG
A-1 CAGCTTCCAGACCAGATTTCATTCTTCAGTGGAAATCCATCAGTTGAAATAGTTCATGGTATTATGCACCTATATAAGACAAATAAGATG
P-G ACCTCCTTAAAAGAAGATGTGCGGCGCAGTGCCATGCTGTGTATTCTCACAGTCCCTGCTGCAATGACCAGTCATGACCTTATGAAGTTT
A-1 ACCTCCTTAAAAGAAGATGTGCGGCGCAGTGCCATGCTGTGTATTCTCACAGTCCCTGCTGCAATGACCAGTCATGACCTTATGAAGTTT
P-G GTTGCCCCATTTAACGAAGTAATTGAACAAATGAAAATTATCAGAGACTCTACTCCCAACCAATATATGGTGCTGATAAAGTTTCGTGCA
A-1 GTTGCCCCATTTAACGAAGTAATTGAACAAATGAAAATTATCAGAGACTCTACTCCCAACCAATATATGGTGCTGATAAAGTTTCGTGCA
P-G CAGGCTGATGCGGATAGTTTTTATATGACATGCAATGGCCGCCAGTTCAACTCAATAGAAGATGACGTTTGCCAGCTAGTGTATGTGGAA
A-1 CAGGCTGATGCGGATAGTTTTTATATGACATGCAATGGCCGCCAGTTCAACTCAATAGAAGATGACGTTTGCCAGCTAGTGTATGTGGAA
P-G AGAGCTGAAGTGCTCAAATCTGAAGATGGCGCCAGCCTCCCAGTGATGGACCTGACTGAACTCCCCAAGTGCACGGTGTGTCTGGAGCGC
A-1 AGAGCTGAAGTGCTCAAATCTGAAGATGGCGCCAGCCTCCCAGTGATGGACCTGACTGAACTCCCCAAGTGCACGGTGTGTCTGGAGCGC
P-G ATGGACGAGTCTGTGAATGGCATCCTCACAACGTTATGTAACCACAGCTTCCACAGCCAGTGTCTACAGCGCTGGGACGATACCACGTGT
A-1 ATGGACGAGTCTGTGAATGGCATCCTCACAACGTTATGTAACCACAGCTTCCACAGCCAGTGTCTACAGCGCTGGGACGATACCACGTGT
P-G CCTGTTTGCCGGTACTGTCAAACGCCCGAGCCAGTAGAAGAAAATAAGTGTTTTGAGTGTGGTGTTCAGGAAAATCTTTGGATTTGTTTA
A-1 CCTGTTTGCCGGTACTGTCAAACGCCCGAGCCAGTAGAAGAAAATAAGTGTTTTGAGTGTGGTGTTCAGGAAAATCTTTGGATTTGTTTA
P-G ATATGCGGCCACATAGGATGTGGACGGTATGTCAGTCGACATGCTTATAAGCACTTTGAGGAAACGCAGCACACGTATGCCATGCAGCTT
A-1 ATATGCGGCCACATAGGATGTGGACGGTATGTCAGTCGACATGCTTATAAGCACTTTGAGGAAACGCAGCACACGTATGCCATGCAGCTT
P-G ACCAACCATCGAGTCTGGGACTATGCTGGAGATAACTATGTTCATCGACTGGTTGCAAGTAAAACAGATGGAAAAATAGTACAGTATGAA
A-1 ACCAACCATCGAGTCTGGGACTATGCTGCAGATAACTATGTTCATCGACTGGTTGCAAGTAAAACAGATGGAAAAATAGTACAGTATGAA
P-G TGTGAGGGGGATACTTGCCAGGAAGAGAAAATAGATGCCTTACAGTTAGAGTATTCATATTTACTAACAAGCCAGCTGGAATCTCAGCGA
A-1 TGTGAGGGGGATACTTGCCAGGAAGAGAAAATAGATGCCTTACAGTTAGAGTATTCATATTTACTAACAAGCCAGCTGGAATCTCAGCGA
P-G ATCTACTGGGAAAACAAGATAGTTCGGATAGAGAAGGACACAGCAGAGGAAATTAACAACATGAAGACCAAGTTTAAAGAAACAATTGAG
A-1 ATCTACTGGGAAAACAAGATAGTTCGGATAGAGAAGGACACAGCAGAGGAAATTAACAACATGAAGACCAAGTTTAAAGAAACAATTGAG
P-G AAGTGTGATAATCTAGAGCACAAACTAAATGATCTCCTAAAAGAAAAGCAGTCTGTGGAAAGAAAGTGCACTCAGCTAAACACAAAAGTG
A-1 AAGTGTGATAATCTAGAGCACAAACTAAATGATCTCCTAAAAGAAAAGCAGTCTGTGGAAAGAAAGTGCACTCAGCTAAACACAAAAGTG
P-G GCCAAACTCACCAACGAGCTCAAAGAGGAGCAGGAAATGAACAAGTGTTTGCGAGCCAACCAAGTCCTCCTGCAGAACAAGCTAAAAGAG
A-1 GCCAAACTCACCAACGAGCTCAAAGAGGAGCAGGAAATGAACAAGTGTTTGCGAGCCAACCAAGTCCTCCTGCAGAACAAGCTAAAAGAG
P-G GAGGAGAGGGTGCTGAAGGAGACCTGTGACCAAAAAGATCTGCAGATCACCGAGATCCAGGAGCAGCTGCGTGACGTCATGTTCTACCTG
A-1 GAGGAGAGGGTGCTGAAGGAGACCTGTGACCAAAAAGATCTGCAGATCACCGAGATCCAGGAGCAGCTGCGTGACGTCATGTTCTACCTG
P-G GAGACACAGCAGAAGATCAACCATCTGCCTGCCGAGACCCGGCAGGAAATCCAGGAGGGACAGATCAACATCGCCATGGCCTCGGCCTCG
A-1 GAGACACAGCAGAAGATCAACCATCTGCCTGCCGAGACCCGGCAGGAAATCCAGGAGGGACAGATCAACATCGCCATGGCCTCGGCCTCG
P-G AGCCCTGCCTCTTCGGGGGGCAGTGGGAAGTTGCCCTCCAGGAAGGGCCGCAGCAAGAGGGGCAAGTGA
A-1 AGCCCTGCCTCTTCGGGGGGCAGTGGGAAGTTGCCCTCCAGGAAGGGCCGCAGCAAGAGGGGCAAGTGA
PTPN11
P-G ATGACATCGCGGAGATGGTTTCACCCAAATATCACTGGTGTGGAGGCAGAAAACCTACTGTTGACAAGAGGAGTTGATGGCAGTTTTTTG
A-1 ATGACATCGCGGAGATGGTTTCACCCAAATATCACTGGTGTGGAGGCAGAAAACCTACTGTTGACAAGAGGAGTTGATGGCAGTTTTTTG
P-G GCAAGGCCTAGTAAAAGTAACCCTGGAGACTTCACACTTTCCGTTAGAAGAAATGGAGCTGTCACCCACATCAAGATTCAGAACACTGGT
A-1 GCAAGGCCTAGTAAAAGTAACCCTGGAGACTTCACACTTTCCGTTAGAAGAAATGGAGCTGTCACCCACATCAAGATTCAGAACACTGGT
P-G GATTACTATGACCTGTATGGAGGGGAGAAATTTGCCACTTTGGCTGAGTTGGTCCAGTATTACATGGAACATCACGGGCAATTAAAAGAG
A-1 GATTACTATGACCTGTATGGAGGGGAGAAATTTGCCACTTTGGCTGAGTTGGTCCAGTATTACATGGAACATCACGGGCAATTAAAAGAG
P-G AAGAATGGAGATGTCATTGAGCTTAAATATCCTCTGAACTGTGCAGATCCTACCTCTGAAAGGTGGTTTCATGGACATCTCTCTGGGAAA
A-1 AAGAATGGAGATGTCATTGAGCTTAAATATCCTCTGAACTGTGCAGATCCTACCTCTGAAAGGTGGTTTCATGGACATCTCTCTGGGAAA
P-G GAAGCAGAGAAATTATTAACTGAAAAAGGAAAACATGGTAGTTTTCTTGTACGAGAGAGCCAGAGCCACCCTGGAGATTTTGTTCTTTCT
A-1 GAAGCAGAGAAATTATTAACTGAAAAAGGAAAACATGGTAGTTTTCTTGTACGAGAGAGCCAGAGCCACCCTGGAGATTTTGTTCTTTCT
P-G GTGCGCACTGGTGATGACAAAGGGGAGAGCAATGACGGCAAGTCTAAAGTGACCCATGTTATGATTCGCTGTCAGGAACTGAAATACGAC
A-1 GTGCGCACTGGTGATGACAAAGGGGAGAGCAATGACGGCAAGTCTAAAGTGACCCATGTTATGATTCGCTGTCAGGAACTGAAATACGAC
P-G GTTGGTGGAGGAGAACGGTTTGATTCTTTGACAGATCTTGTGGAACATTATAAGAAGAATCCTATGGTGGAAACATTGGGTACAGTACTA
A-1 GTTGGTGGAGGAGAACGGTTTGATTCTTTGACAGATCTTGTGGAACATTATAAGAAGAATCCTATGGTGGAAACATTGGGTACAGTACTA
P-G CAACTCAAGCAGCCCCTTAACACGACTCGTATAAATGCTGCTGAAATAGAAAGCAGAGTTCGAGAACTAAGCAAATTAGCTGAGACCACA
A-1 CAACTCAAGCAGCCCCTTAACACGACTCGTATAAATGCTGCTGAAATAGAAAGCAGAGTTCGAGAACTAAGCAAATTAGCTGAGACCACA
P-G GATAAAGTCAAACAAGGCTTTTGGGAAGAATTTGAGACACTACAACAACAGGAGTGCAAACTTCTCTACAGCCGAAAAGAGGGTCAAAGG
A-1 GATAAAGTCAAACAAGGCTTTTGGGAAGAATTTGAGACACTACAACAACAGGAGTGCAAACTTCTCTACAGCCGAAAAGAGGGTCAAAGG
P-G CAAGAAAACAAAAACAAAAATAGATATAAAAACATCCTGCCCTTTGATCATACCAGGGTTGTCCTACACGATGGTGATCCCAATGAGCCT
A-1 CAAGAAAACAAAAACAAAAATAGATATAAAAACATCCTGCCCTTTGATCATACCAGGGTTGTCCTACACGATGGTGATCCCAATGAGCCT
P-G GTTTCAGATTACATCAATGCAAATATCATCATGCCTGAATTTGAAACCAAGTGCAACAATTCAAAGCCCAAAAAGAGTTACATTGCCACA
A-1 GTTTCAGATTACATCAATGCAAATATCATCATGCCTGAATTTGAAACCAAGTGCAACAATTCAAAGCCCAAAAAGAGTTACATTGCCACA
P-G CAAGGCTGCCTGCAAAACACGGTGAATGACTTTTGGCGGATGGTGTTCCAAGAAAACTCCCGAGTGATTGTCATGACAACGAAAGAAGTG
A-1 CAAGGCTGCCTGCAAAACACGGTGAATGACTTTTGGCGGATGGTGTTCCAAGAAAACTCCCGAGTGATTGTCATGACAACGAAAGAAGTG
P-G GAGAGAGGAAAGAGTAAATGTGTCAAATACTGGCCTGATGAGTATGCTCTAAAAGAATATGGCGTCATGCGTGTTAGGAACGTCAAAGAA
A-1 GAGAGAGGAAAGAGTAAATGTGTCAAATACTGGCCTGATGAGTATGCTCTAAAAGAATATGGCGTCATGCGTGTTAGGAACGTCAAAGAA
P-G AGCGCCGCTCATGACTATACGCTAAGAGAACTTAAACTTTCAAAGGTTGGACAAGGGAATACGGAGAGAACGGTCTGGCAATACCACTTT
A-1 AGCGCCGCTCATGACTATACGCTAAGAGAACTTAAACTTTCAAAGGTTGGACAAGGGAATACGGAGAGAACGGTCTGGCAATACCACTTT
P-G CGGACCTGGCCGGACCACGGCGTGCCCAGCGACCCTGGGGGCGTGCTGGACTTCCTGGAGGAGGTGCACCATAAGCAGGAGAGCATCATG
A-1 CGGACCTGGCCGGACCACGGCGTGCCCAGCGACCCTGGGGGCGTGCTGGACTTCCTGGAGGAGGTGCACCATAAGCAGGAGAGCATCATG
P-G GATGCAGGGCCGGTCGTGGTGCACTGCAGTGCTGGAATTGGCCGGACAGGGACGTTCATTGTGATTGATATTCTTATTGACATCATCAGA
A-1 GATGCAGGGCCGGTCGTGGTGCACTGCAGTGCTGGAATTGGCCGGACAGGGACGTTCATTGTGATTGATATTCTTATTGACATCATCAGA
P-G GAGAAAGGTGTTGACTGCGATATTGACGTTCCCAAAACCATCCAGATGGTGCGGTCTCAGAGGTCAGGGATGGTCCAGACAGAAGCACAG
A-1 GAGAAAGGTGTTGACTGCGATATTGACGTTCCCAAAACCATCCAGATGGTGCGGTCTCAGAGGTCAGTGATGGTCCAGACAGAAGCACAG
P-G TACCGATTTATCTATATGGCGGTCCAGCATTATATTGAAACACTACAGCGCAGGATTGAAGAAGAGCAGAAAAGCAAGAGGAAAGGGCAC
A-1 TACCGATTTATCTATATGGCGGTCCAGCATTATATTGAAACACTACAGCGCAGGATTGAAGAAGAGCAGAAAAGCAAGAGGAAAGGGCAC
P-G GAATATACAAATATTAAGTATTCTCTAGCGGACCAGACGAGTGGAGATCAGAGCCCTCTCCCGCCTTGTACTCCAACGCCACCCTGTGCA
A-1 GAATATACAAATATTAAGTATTCTCTAGCGGACCAGACGAGTGGAGATCAGAGCCCTCTCCCGCCTTGTACTCCAACGCCACCCTGTGCA
P-G GAAATGAGAGAAGACAGTGCTAGAGTCTATGAAAACGTGGGCCTGATGCAACAGCAGAAAAGTTTCAGATGA
A-1 GAAATGAGAGAAGACAGTGCTAGAGTCTATGAAAACGTGGGCCTGATGCAACAGCAGAAAAGTTTCAGATGA

## Slide 5
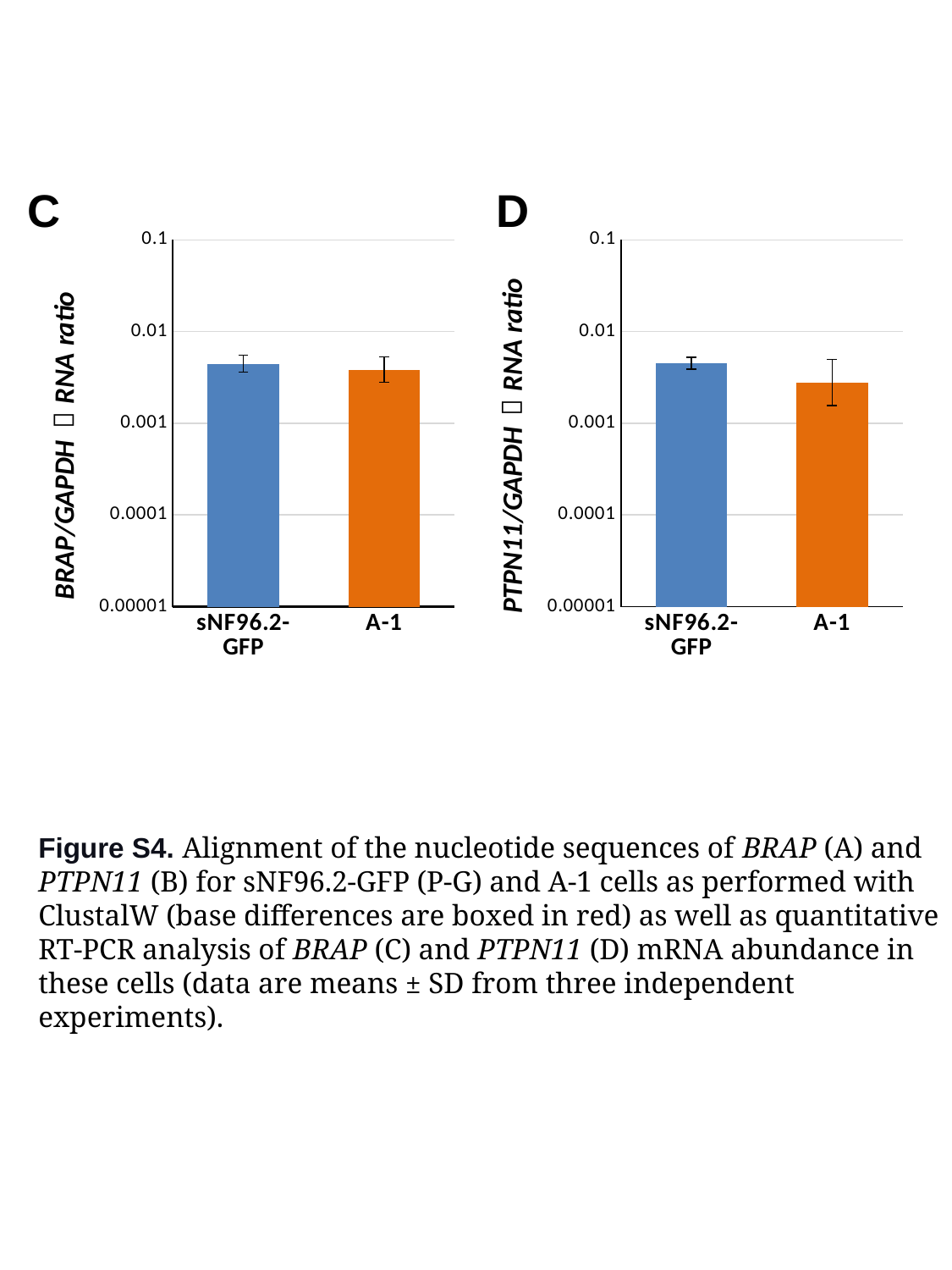

D
C
### Chart
| Category | |
|---|---|
| sNF96.2-GFP | 0.004456107 |
| A-1 | 0.003843583 |
### Chart
| Category | |
|---|---|
| sNF96.2-GFP | 0.004528767 |
| A-1 | 0.00277493 |Figure S4. Alignment of the nucleotide sequences of BRAP (A) and PTPN11 (B) for sNF96.2-GFP (P-G) and A-1 cells as performed with ClustalW (base differences are boxed in red) as well as quantitative RT-PCR analysis of BRAP (C) and PTPN11 (D) mRNA abundance in these cells (data are means ± SD from three independent experiments).
